# Spread of Meropenem-Resistant *Streptococcus pneumoniae* Serotype 15A-ST63 Clone in Japan, 2012–2014

**DOI:** 10.3201/eid2402.171268

**Published:** 2018-02

**Authors:** Satoshi Nakano, Takao Fujisawa, Yutaka Ito, Bin Chang, Yasufumi Matsumura, Masaki Yamamoto, Miki Nagao, Shigeru Suga, Makoto Ohnishi, Satoshi Ichiyama

**Affiliations:** Kyoto University Graduate School of Medicine, Kyoto, Japan (S. Nakano, Y. Matsumura, M. Yamamoto, M. Nagao, S. Ichiyama);; National Hospital Organization Mie National Hospital, Tsu, Japan (T. Fujisawa, S. Suga);; Nagoya City University Graduate School of Medical Science, Nagoya, Japan (Y. Ito);; National Institute of Infectious Diseases, Tokyo, Japan (B. Chang, M. Ohnishi)

**Keywords:** *Streptococcus pneumoniae*, meropenem resistance, antimicrobial resistance, serotype 15A, ST63, PCV7, PCV13, bacteria, Japan, vaccines, vaccination, pneumococcal conjugate vaccine

## Abstract

After the introduction of pneumococcal conjugate vaccines, the incidence of pneumococcal infections due to meropenem-resistant serotype 15A-ST63 strains increased in Japan. By using whole-genome sequencing and comparing sequences with those of clones from the United Kingdom, the United States, and Canada, we clarified the traits of the serotype 15A-ST63 clone. Our analysis revealed that the meropenem-resistant serotype 15A-ST63 strains from Japan originated from meropenem-susceptible strains from Japan. Recombination site prediction analysis showed that the meropenem-resistant strain-specific recombination regions included the *pbp1a* and *pbp2b* regions. A detailed analysis of the composition of these genes indicated that resistance seems to be caused by *pbp1a* recombination. The *pbp1a* gene in meropenem-resistant isolates was identical to that in multidrug (including meropenem)–resistant serotype 19A-ST320 pneumococci, which have spread in the United States. The global spread of pneumococci of this lineage is noteworthy because serotype 15A is not included in the currently used 13-valent pneumococcal conjugate vaccine.

*Streptococcus pneumoniae* is a common pathogen that causes various types of bacterial infections, such as pneumonia, otitis media, occult bacteremia, and meningitis (*1*). *S. pneumoniae* is enclosed in a complex polysaccharide capsule that can be used to classify strains into serotypes. So far, at least 92 structurally and serologically distinct serotypes have been recognized (*2*). To prevent invasive pneumococcal diseases, vaccines currently in use in various regions of the world are 7-, 10-, and 13-valent pneumococcal conjugate vaccines (PCVs) that target a subset of the serotypes. Although these vaccines have decreased the total number of cases of invasive pneumococcal disease, they have also caused a shift in serotype (i.e., increased rate of identification of non-PCV serotype pneumococci) in areas where the vaccines have been introduced (*3*–*6*). Public health officials are concerned about the increased incidence of non-PCV serotype pneumococcal infections and the spread of resistant strains that are not covered by the currently used PCVs.

In Japan when PCV13 was in use (2012–2014), we conducted a nationwide pneumococcal infection surveillance study among children (*7*). We observed an increase in multidrug (penicillin, macrolide, and meropenem)–resistant pneumococcal isolates of serotype 15A and sequence type (ST) 63 (serotype 15A-ST63), which is not covered by PCV13. Similarly, several surveillance studies in other countries also reported an increase in serotype 15A-ST63 pneumococcal infections, including infections caused by penicillin-resistant strains (*8*–*16*) but not meropenem-resistant serotype 15A pneumococcal strains. A pneumococcal strain of serotype 15A-ST63 from Sweden was submitted as a resistant clone to the Pneumococcal Molecular Epidemiology Network (PMEN) collection (Sweden^15A^-25, ATCC BAA-661) (*17*); however, the penicillin MIC for Sweden^15A^-25 differs from that for serotype 15A-ST63 from Japan. Although the submitted PMEN strain is slightly resistant to penicillin (MIC 0.064), most serotype 15A-ST63 isolates from Japan are more resistant to penicillin. Although the introduction of PCVs is thought to have driven the increase in non-PCV pneumococcal infections, the mechanism by which the strains have become resistant remains unclear.

In general, pneumococcal resistance to penicillin and cephalosporins (including carbapenems) is caused by mutations in penicillin binding proteins (PBPs), especially PBPs 1a, 2b, and 2x (*18*). Although PBP genes of sensitive pneumococci are well conserved, PBPs of resistant isolates are encoded by highly variable genes containing sequence blocks that are generally referred to as mosaic genes; these genes are generated by recombination events (*19*–*21*). Therefore, tracking the sequences of PBP genes is useful for predicting resistance to antimicrobial drugs and following pneumococcal epidemiologic trends. In various countries, meropenem is widely used to treat severe infectious diseases; however, infectious diseases caused by pathogens resistant to meropenem (carbapenems) have been increasing (*22*). Although carbapenem resistance has been noted mainly in *Enterobacteriaceae*, resistance in other pathogens, including *S. pneumoniae*, should be noted because such broad-spectrum antimicrobial drugs are often used empirically.

Our aim with this study was to clarify the genetic characteristics of meropenem-resistant serotype 15A-ST63 strains isolated in Japan. In addition, using whole-genome sequencing data, we mapped the evolution of the clone by revealing the genetic associations among drug-resistant serotype 15A-ST63 pneumococcus isolates from different areas of the world (Japan, United Kingdom, United States, and Canada).

## Materials and Methods

### Bacterial Isolates

From January 2012 through December 2014, we conducted a nationwide, prospective surveillance study among children in Japan with and without invasive pneumococcal disease (*7*). From 154 medical institutions, we collected isolates from patients with (343 isolates) and without (286 isolates) invasive pneumococcal disease. Among these 629 isolates, we obtained 52 serotype 15A-ST63 isolates, including 35 meropenem-nonsusceptible (MEM-NS) isolates and 17 meropenem-susceptible (MEM-S) isolates. With regard to MEM-NS non–serotype 15A isolates, we obtained 66 isolates comprising 8 serotypes (6A, 6B, 6D, 15B/C, 19A, 19F, 23F, and 35B) and nontypeable serotypes. Following the 2008 Clinical and Laboratory Standards Institute guidelines (*23*), we performed susceptibility testing for meropenem by using the broth microdilution method and defined the MEM-NS MIC as >0.5 mg/L and the MEM-S MIC as <0.25 mg/L.

### Whole-Genome Sequencing

Of the isolates described above, we obtained read data for 24 MEM-NS serotype 15A-ST63 isolates, 10 MEM-S serotype 15A-ST63 isolates, and 32 MEM-NS non–serotype 15A (6A, 6B, 6D, 15B/C, 19A, 19F, 23F, 35B, and untypeable) isolates (Technical Appendix Table 1). To achieve a uniform distribution of regions and times where and when the isolates were identified, we randomly selected 24 MEM-NS serotype 15A-ST63, 10 MEM-S serotype 15A-ST63, and 32 MEM-NS non–serotype 15A isolates from the isolate repository (Table 1). In addition, we obtained read data for the PMEN 15A-25 strain (Sweden^15A^-25, ATCC BAA-662). We used a QIAamp DNA Mini Kit (QIAGEN, Hilden, Germany) to extract total genomic DNA from each bacterial isolate and the Nextera XT DNA Library Preparation Kit (Illumina, San Diego, CA, USA) to prepare libraries for sequencing. We multiplexed and sequenced the samples on an Illumina MiSeq for 600 cycles (2 × 300-bp paired-end).

**Table 1:** Pneumococcal isolates from Japan, 2012–2014, and MICs for penicillin, meropenem, and erythromycin

Serotype and sequence type	No. isolates	MIC, μg/mL									
		Penicillin				Meropenem			Erythromycin		
		<0.06	0.12–1.0	>2.0		<0.06	>0.5		<0.25	0.5	>1.0
15A											
63	34	0	13	21		10	24		0	0	34
15B/C											
83	2	0	0	2		0	2		0	0	2
3934	1	0	1	0		0	1		0	0	1
19A											
3111	7	0	4	3		0	7		0	0	7
320	4	0	0	4		0	4		0	0	4
19F											
236	2	0	0	2		0	2		0	0	2
115	1	0	0	1		0	1		0	0	1
23F											
242	1	0	0	1		0	1		0	0	1
35B											
558	8	0	5	3		0	8		2	0	6
6A											
2756	1	0	0	1		0	1		0	0	1
6B											
9335	1	0	0	1		0	1		0	0	1
6D											
282	1	0	0	1		0	1		0	0	1
Untypeable											
7502	1	0	0	1		0	1		0	0	1
4845	1	0	0	1		0	1		0	0	1
10253	1	0	0	1		0	1		0	0	1
Total	66	0	23	43		10	56		2	0	64

### Comparing Genomic Data 

To compare the genomic characteristics of serotype 15A-ST63 isolates from Japan with those of isolates from the United Kingdom, the United States, and Canada, we downloaded whole-genome read data from the Sequence Read Archive database (http://www.ncbi.nlm.nih.gov/sra/) (Technical Appendix Table 1) (*5*,*8*,*24*,*25*). To confirm that the data were from serotype 15A-ST63 isolates, we conducted a de novo assembly by using SPAdes (*26*). The contigs were analyzed by using BLAST+ (*27*) to verify the presence of the pneumococcal serotype 15A–specific region (Technical Appendix Table 5) (*5*), followed by multilocus sequence typing (MLST) (http://pubmlst.org/spneumoniae/), which used the extracted subsequences of each allele from the contigs. We used the draft genome data that contained serotype 15A–specific genes from ST63 isolates for the subsequent analysis.

### Phylogenomic Analyses 

To create a phylogenic tree, we used Genealogies Unbiased By recomBinations In Nucleotide Sequences (Gubbins) (*28*), which identifies recombination sites and constructs a phylogenic tree based on the putative point mutations outside of the regions. First, we created 2 phylogenic trees. Although there were no ST63 isolates or isolates that were closely related to ST63 with non–serotype 15A, to prove that there were no serotype switch events from non–serotype 15A to serotype 15A, we created the first tree with isolates from Japan only. To reveal the genetic associations among serotype 15A-ST63 isolates from Japan and elsewhere around the world that are increasing in incidence, we created the second tree with only serotype 15A-ST63 isolates from Japan and other regions in the world. After obtaining the second phylogenic tree, to predict the recombination sites that caused meropenem resistance, we created an additional phylogenic tree by using the isolates that were clustered into the same clade that included MEM-NS and MEM-S serotype 15A-ST63 isolates.

### PBP Profiles, Antimicrobial Resistance Genes, and Pilus Detection

To compare the sequences of the transpeptidase regions of *pbp1a*, *2b*, and *2x* of all isolates, we extracted each PBP transpeptidase region from all obtained contigs by using BLAST+. We allocated PBP transpeptidase type numbers to these contigs by using previous PBP sequence data from the United States (*5*,*29*–*31*). To predict a causal PBP transpeptidase type for meropenem resistance in serotype 15A-ST63 isolates from Japan, we identified recombination sites within these serotype 15A isolates by using Gubbins. In addition, we identified the presence of the *ermB*, *ermTR*, *mefA*, *mefE*, *tetM*, *tetO*, *rrgA-1* (pili1), and *pitB-1* (pili2) genes and searched for mutations within the *folA* and *folP* genes by using the assembled contigs (*5*). Details of the genomic analysis process are described in the online Technical Appendix.

## Results

### Whole-Genome Sequencing Statistics

The sequencing statistics are shown in Technical Appendix Table 2. With use of the 2,078,953-bp *S. pneumoniae* G54 chromosome (reference sequence GenBank accession no. NC_011072.1), serotype 15A-ST63 and PMEN15A-25 isolate genomes were sequenced at an average depth (± SD) of 49.33 (± 9.65) and an average coverage of 95.60% (± 3.75%). The average numbers of contigs and *N*_50_ (bp) of isolates from Japan sequenced in this study were 109.2 (SD ± 42.2) and 70,819 (SD ± 10,701), respectively.

### Phylogenomics 

The phylogenic tree created by using all Japan and global isolates classified these isolates into 11 clusters (Technical Appendix Figure 1). All of the Japan serotype 15A isolates were included in the same cluster, and none of the non–serotype 15A isolates were included in this cluster. This fact indicated that none of the non–serotype 15A isolates in this analysis seemed to be the origin of the MEM-NS serotype 15A-ST63 isolates.

**Figure 1:**
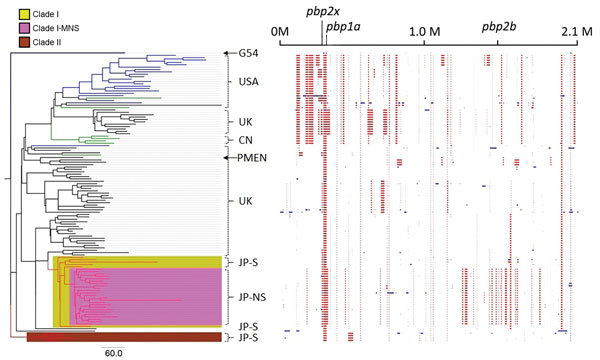
Phylogenic tree and predicted recombination sites created in Genealogies Unbiased By recomBinations In Nucleotide Sequences (*28*) by using all Japan and global serotype 15A-ST63 pneumococcal isolates. Branch colors in the tree indicate where the isolates were collected: red, Japan; black, United Kingdom; blue, United States; green, Canada. The column on the right of the tree indicates the main region from which the isolates were derived, meropenem susceptibility, and isolate names. The phylogenic tree was created by using *Streptococcus pneumoniae* G54 as an outgroup isolate. Clade I consists of only Japan serotype 15A-ST63 isolates; clade I-MNS consists of only Japan meropenem-nonsusceptible serotype 15A-ST63 isolates; clade II consists of the rest of the Japan meropenem-susceptible serotype 15A-ST63 isolates that are not included in clade I. The block chart on the right shows the predicted recombination sites in each isolate. Blue blocks are unique to a single isolate; red blocks are shared by multiple isolates. All isolates shaded in pink are meropenem nonsusceptible. Arrows indicate reference strains *S. pneumoniae* G54 and PMEN 15A-25. Scale bar indicates nucleotide substitutions per site; CN, Canada; G54, *S. pneumoniae* G54; M, million base pairs; JP-NS, Japan meropenem nonsusceptible; JP-S, Japan meropenem susceptible; PMEN, Pneumococcal Molecular Epidemiology Network; ST, sequence type; UK, United Kingdom; USA, United States.

The phylogenic tree created by using all Japan and global serotype 15A-ST63 isolates revealed the presence of 2 serotype 15A-ST63–specific clades (clades I and II) from Japan (Figure 1). Clade I included the subclade clade I-MNS, which consisted of all 24 MEM-NS serotype 15A-ST63 isolates. This result indicated that the Japan MEM-NS isolates originated from Japan MEM-S isolates. Four other Japan MEM-S serotype 15A-ST63 isolates were classified into clade II. With some exceptions, the global isolates were clustered according to the areas where the isolates were recovered.

We identified 52 genes that were specific to the subclade clade I-MNS. These genes did not exist in Japan MEM-S serotype 15A-ST63 isolates but were found in the Japan MEM-NS isolates (Technical Appendix Methods, Table 4).

### PBP Recombination Sites that Could Cause Meropenem Resistance

**Figure 2:**
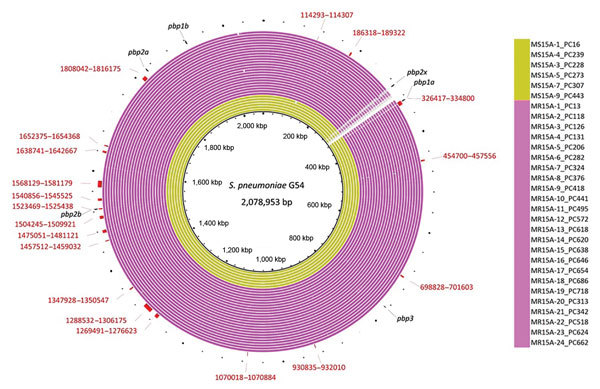
Genomic similarities to *Streptococcus pneumoniae* G54 (reference sequence GenBank accession no. NC_011072.11) and Japan meropenem-nonsusceptible serotype 15A-ST63 isolate-specific recombination sites that were obtained in Genealogies Unbiased By recomBinations In Nucleotide Sequences (*28*) by using all clade I and clade I-MNS isolates. Colored segments indicate >95% similarity; gray segments indicate >90% similarity by BLAST (*27*) comparison between each isolate genome and *S. pneumoniae* G54. The outside red bars indicate the recombination sites that were specific to meropenem-nonsusceptible serotype 15A-ST63 isolates and identified in all of these isolates. Red numbers indicate the sequence coordinates of the recombination sites when *S. pneumoniae* G54 was used. Outside short black lines indicate each penicillin binding protein region. MR, Japan meropenem nonsusceptible; MS, Japan meropenem susceptible; ST, sequence type.

Using all of the clade I isolates, we predicted the recombination sites that caused meropenem resistance in the MEM-NS serotype 15A-ST63 isolates from Japan. This analysis revealed 19 recombination sites that were specific to all MEM-NS isolates (Figure 2; Technical Appendix Figure 2). One of these recombination sites included the whole *pbp1a* gene, and another overlapped with a part of the nucleotide sequence of *pbp2b*. The recombination site covering *pbp1a* included 8,384 bp (positions 326417–334800 of the reference strain *S. pneumoniae* G54) (Technical Appendix Figure 3). All MEM-NS serotype 15A-ST63 isolates had the same nucleotide sequence of *pbp1a* as those of MEM-NS serotype 19A, 19F, 23F, 6A, 6B, and nontypeable isolates from Japan. The recombination site that overlapped with the *pbp2b* gene was a 1,970-bp region (positions 1523469–1525438 of the reference strain *S. pneumoniae* G54) (Technical Appendix Figure 3). Because of this recombination, a portion of the nucleotide sequence of *pbp2b* was replaced with the current sequence, resulting in development of a novel MEM-NS serotype 15A-ST63 *pbp2b* gene that was not found in other isolates in this study or in the public database.

### Comparison of the PBP Profiles and 3 Conserved Amino Acid Motifs

#### pbp1a

All Japan MEM-NS serotype 15A-ST63 isolates had type 13 *pbp1a*, which has been identified mainly in multidrug-resistant serotype 19A and 19F isolates from the United States (Table 2; Technical Appendix Tables 1, 6) (*5*). Of 32 Japan MEM-NS non–serotype 15A isolates, 18 also had type 13 *pbp1a*. These 18 isolates included 10 serotype 19A isolates, 3 serotype 19F isolates, and isolates of 5 other serotypes. All Japan MEM-S serotype 15A-ST63 isolates had type 24 *pbp1a,* which was identified in the PMEN15A-25 isolate and in penicillin-intermediate-resistant and MEM-S serotype 15A isolates in the United States. Of 86 global isolates, 74 also had type 24 *pbp1a* and the other 12 had novel *pbp1a* genes. All 24 Japan MEM-NS serotype 15A-ST63 isolates had the same SSMK motif (Table 3). All 10 Japan MEM-S serotype 15A-ST63 isolates had the STMK motif.

**Table 2:** Penicillin binding protein profile of *Streptococcus pneumonaie* serotype 15A-ST63 isolates from Japan, 2012–2014*

Clone (no.)	Penicillin binding protein profile			
	*pbp1a* (no.)	*pbp2b* (no.)	*pbp2x* (no.)	*pbp1a:pbp2b:pbp2x* (no.)
MEM-S-15A-ST63 (10)	24 (10)	27 (10)	43 (5), 28 (3), 112 (1), new1 (1)	24:27:43 (5), 24:27:28 (3), 24:27:112 (1), 24:27:new1 (1)
MEM-NS-15A-ST63 (24)	13 (24)	new1 (24)	43 (22), new3 (1), new6 (1)	13:new1:43 (22), 13:new1:new3 (1), 13:new1:new6 (1)
MEM-NS-19A-ST320 (4)	13 (4)	11 (4)	16 (4)	13:11:16 (4)

*MEM-NS, meropenem-nonsusceptible; MEM-S, meropenem-susceptible; ST, sequence type.

**Table 3:** PBP 1a, 2b, and 2x transpeptidase conserved amino acid motif profile of *Streptococcus pneumonaie* isolates from Japan, 2012–2014*

Clone (no.)	Sequences of conserved amino acid motifs of PBPs										
*pbp1a*				*pbp2b*				*pbp2x*		
SXXK (no.)	SXN (no.)	KTG (no.)		SXXK (no.)	SXN (no.)	KSG (no.)		SXXK (no.)	SXN (no.)	KSG (no.)
MEM-S-15A-ST63 (10)	STMK (10)	SRN (10)	KTG (10)		SVVK (10)	SSNA (10)	KTG (10)		SPMK (1), STMK (3), SAMK (6)	HSSN (10)	VKSG (6), LKSG (4)
MEM-NS-15A-ST63 (24)	SSMK (24)	SRN (24)	KTG (24)		SVVK (24)	SSNA (24)	KTG (24)		SAMK (22), SAFK (2)	HSSN (24)	VKSG (24)
MEM-NS-non15A (32)	SSMK (29), SAMK (3)	SRN (32)	KTG (32)		SVVK (32)	SSNA (32)	KTG (32)		SAMK (32)	HSSN (32)	VKSG (32)
PMEN15A-25 (1)	STMK (1)	SRN (1)	KTG (1)		SVVK (1)	SSNA (1)	KTG (1)		STMK (1)	HSSN (1)	LKSG (1)

*MEM-NS, meropenem-nonsusceptible; MEM-S, meropenem-susceptible; PBP, penicillin binding protein; PMEN, Pneumococcal Molecular Epidemiology Network; ST, sequence type.

#### pbp2b

The Japan MEM-NS serotype 15A-ST63 isolates had a specific novel *pbp2b* type that was not found in the US *pbp2b* profile list (Table 2; Technical Appendix Tables 1, 7) (*5*). In the reference *pbp2b* type database, type 74 *pbp2b* was the closest to the novel type from Japan, which possessed 5 aa sequence mutations. Japan MEM-NS non–serotype 15A isolates showed 8 *pbp2b* types, and the types differed from each other mainly by serotype. All Japan MEM-S serotype 15A-ST63 isolates showed type 27 *pbp2b,* which was identified in penicillin-intermediate-resistant and MEM-S serotype 15A isolates in the United States. Of 86 global isolates, 83 also had type 27 *pbp*2b. All sequenced isolates from Japan, including MEM-NS and MEM-S serotype 15A-ST63 isolates and MEM-NS non–serotype 15A isolates, had the same SVVK, SSN, and KTG motifs (Table 3).

#### pbp2x

In contrast to the *pbp1a* and *pbp2b* type profiles, the *pbp2x* type profile showed a more complicated distribution. The most prevalent *pbp2x* type in the Japan MEM-NS serotype 15A-ST63 isolates was type 43 (22/24 isolates), and 5 of 10 Japan MEM-S serotype 15A-ST63 isolates also had the type 43 *pbp*2x gene (Table 2; Technical Appendix Tables 1, 8). This type was identified in a serotype 19F-CC177 isolate from the United States that was resistant to penicillin and susceptible to meropenem (*5*). The other 2 MEM-NS serotype 15A-ST63 isolates each had a novel *pbp*2x type. Among the global isolates, the most prevalent *pbp*2x type was type 28 (55/86 isolates), followed by type 35 (12/86) and type 179 (12/86). Similar to the *pbp*2b type, the Japan MEM-NS non–serotype 15A isolates showed different *pbp2x* types on the basis of their serotypes. The most prevalent SXXK motif in the Japan MEM-NS serotype 15A-ST63 and MEM-S serotype 15A-ST63 isolates was SAMK (Table 3). All sequenced isolates from Japan had the same SSN and KSG motifs.

### Antimicrobial Resistance Genes and Pilus Determinants

All serotype 15A-ST63 isolates from Japan had *tetM* and *ermB* genes and were negative for the *ermTR*, *tetO*, *mefA*, and *mefE* genes; *folA* mutation; and *folP* insertion (Technical Appendix Table 3). Only 1 of 34 Japan serotype 15A-ST63 isolates had a deletion of 2 nt at codon 339 in *tetM*, generating a premature stop codon; resistance gene prevalence did not differ between MEM-S and MEM-NS serotype 15A-ST63 isolates from Japan. The same deletion was identified in 12 of the isolates from the United Kingdom and in PMEN15A-25. In addition, all Japan and global serotype 15A isolates lacked pilus determinants PI-1 and PI-2. With regard to global serotype 15A-ST63 isolates, the profiles of *tetM*, *ermB*, *tetO*, *mefA*, and *mefE* were the same as those of Japan serotype 15A-ST63 isolates: positive for *tetM* and *ermB* and negative for *ermTR*, *tetO*, *mefA*, and *mefE*. However, 16 isolates (5 from the United States and 11 from the United Kingdom) had a *folA* mutation (I100L substitution). All 16 of these isolates also had a *folP* insertion (1–2 codons between bases 168 and 201). An additional 8 isolates (7 from the United States and 1 from Canada) had only the *folP* insertion.

## Discussion

After the introduction of PCVs, serotype 15A pneumococcal infections and colonization increased in many countries (*7*–*16*). According to previous molecular studies, most serotype 15A isolates belonged to ST63, which has been named Sweden^15A^-25 (PMEN15A-25) in the PMEN and shows strong macrolide resistance. The PMEN database and previous studies regarding serotype 15A-ST63 strains indicate that most isolates that were closely related to PMEN15A-25 were susceptible (MIC <0.06 mg/L) or showed intermediate resistance (MIC 0.12–1.0 mg/L) to penicillin (*5*,*9*,*10*,*32*). Although data for meropenem susceptibility of these isolates are limited (*5*,*7*), no studies have demonstrated the meropenem resistance of this strain. Thus, the spread of this strain so far seems to be limited to Japan. However, this finding is of concern for several reasons. One reason is the fact that serotype 15A is not included in the currently used PCV13; therefore, the increased incidence would continue under the current vaccine pressure. In addition, penicillin, meropenem, and macrolide resistance may cause the strain to spread rapidly. In fact, after the introduction of PCV7, multidrug-resistant serotype 19A-CC320/271 spread rapidly and widely in the United States (*33*).

Several previous studies revealed the emergence of serotype-switched new strains that showed resistance to several antimicrobials (*33*–*35*). Most of the mechanisms underlying the emergence of new resistant strains are associated with the recombination of the *cps* region flanking *pbp1a* and *pbp2x*; resistant strains switched their serotypes via recombination of the *cps* region, or susceptible strains gained *pbp1a* and/or *pbp2x* resistance genes with the *cps* region. In the MEM-NS serotype 15A-ST63 isolates investigated in this study, the recombination sites that caused meropenem resistance included the *pbp1a* and *pbp2b* regions but did not include the *cps* region; thus, the serotype switch did not occur. The nucleotide sequence of *pbp1a,* including the transpeptidase region found in MEM-NS serotype 15A-ST63 isolates, was 100% identical to that of the meropenem-resistant serotype 19A-ST320 strain that is prevalent in the United States (type 13 *pbp1a*) (*5*). This serotype 19A-ST320 strain was also recovered in our previous surveillance study in Japan (*7*), and the transpeptidase region of the isolates was the same as that of MEM-NS serotype 15A-ST63 isolates. This finding suggests that the MEM-S serotype 15A-ST63 strain gained the meropenem resistance–related *pbp1a* gene to become the MEM-NS serotype 15A-ST63 strain. Of note, this *pbp1a* type was identified in several Japan MEM-NS serotype isolates, such as 19F, 23F, 6A, 6B, and nontypeable isolates. According to previous PBP profile data from the United States (*5*), type 13 *pbp1a* was found in serotype 19A-ST320 only, and there were no widely spread PBP types across many resistant lineages. 

In Japan, broad-spectrum oral cephalosporin, fluoroquinolones, and macrolides have been frequently prescribed. The inappropriate use of antimicrobial drugs may provide selective pressure and cause the spread of meropenem-resistant strains by the transfer of the meropenem resistance–related *pbp1a* gene.

The Japan MEM-NS serotype 15A-ST63 isolates had a novel, specific *pbp2b* type. The contribution of this *pbp2b* type to meropenem resistance is not clear. One of the MEM-NS serotype 15A-ST63–specific recombination sites overlapped the *pbp2b* region; therefore, this recombination may have caused meropenem resistance. However, all of the MEM-NS and MEM-S serotype 15A-63 isolates had the same sequences in each of 3 conserved amino acid motifs of *pbp2b*. We believe that this result reduced the likelihood that *pbp2b* recombination is associated with meropenem resistance. In addition, we identified 17 other MEM-NS serotype 15A-ST63–specific recombination sites that occurred outside of *pbp1a* and *pbp2b*. It is possible that these recombination events could result in meropenem resistance by non-PBP mutations.

We note the usefulness of PBP typing for predicting drug resistance and tracing the geographic genetic trends in pneumococci. MLST has been widely used in epidemiologic studies of pneumococci to trace genetic trends and to predict serotype switch events that occasionally lead to the development of antimicrobial drug–resistant clones. However, as in our studies, MLST is unable to predict recombination events that occur without a serotype switch, even if the recombination leads to the development of resistance. These facts highlight the value of PBP typing, and these types of data would support future studies of pneumococci.

All analyzed isolates were positive for *ermB* and *tetM*; however, several isolates had a 2-nt deletion in *tetM,* which generated a premature stop codon. This deletion was mainly identified in the UK isolates, and only 1 Japan isolate showed this deletion. This deletion was also identified in PMEN15A-25, which was recovered in Portugal in 1998. Considering that *tetM* generally exists on a transposable element, these results may imply that the origins of *tetM* differ from those of isolates from Japan, the United States, and Europe; *tetM* may exist on different transposable elements in each region, which may have been imported from different sources. In addition, the complete conservation of *ermB* and *tetM* may indicate that these genes contribute to its global spread.

This study had limitations. First, we examined fewer Japan MEM-S than MEM-NS serotype 15A-ST63 isolates, which may have reduced the accuracy of the recombination site prediction. However, the 2 obtained phylogenic trees, one that was constructed by using all isolates of serotype 15A-ST63 and another that was constructed by using only Japan serotype 15A-ST63 isolates, resulted in similar clade Is. Therefore, we believe that the effect of the small number on the result was low. Second, we analyzed serotype 15A-ST63 isolates recovered from only 4 countries with a reference isolate. Future studies that include many isolates from other countries will provide additional insights into the spread and evolution of the serotype 15A-ST63 strains.

In conclusion, MEM-NS serotype 15A-ST63 pneumococci have spread in Japan after the introduction of PCV7 and PCV13. This strain originated from the MEM-S serotype 15A-ST63 strain that was prevalent in Japan; MEM-S serotype 15A-ST63 became MEM-NS serotype 15A-ST63 because of the recombination of the *pbp1a* region. The causative *pbp1a* fragment seemed to have been transferred from the MEM-NS serotype 19A-ST320 strain, and the fragment was identified in many meropenem-resistant serotype isolates in Japan. The Japan and North America serotype 15A-ST63 strains seemed to lack the original *tetM* gene that had a premature stop codon. The global spread of this lineage is noteworthy because serotype 15A is not included in the currently used PCV13. 

Technical Appendix Supplementary methods for study of spread of a meropenem-resistant *Streptococcus pneumoniae* serotype 15A-ST63 clone in Japan, 2012–2014.
