# Correction: Vol. 24, No. 2

**DOI:** 10.3201/eid2406.C52406

**Published:** 2018-06

**Keywords:** errata, erratum, correction

Some data were inaccurate in the text and figures in Spread of Meropenem-Resistant *Streptococcus pneumoniae* Serotype 15A-ST63 Clone in Japan, 2012–2014 (S. Nakano et al.). The article has been corrected online (https://wwwnc.cdc.gov/eid/article/24/2/17-1276_article).

