# Supplementary material for: Spread of Meropenem-Resistant Streptococcus pneumoniae Serotype 15A-ST63 Clone in Japan, 2012–2014
Source: Emerg Infect Dis. 2018 Feb;24(2):275–83. doi: 10.3201/eid2402.171268 (PMC5782878; doi:10.3201/eid2402.171268)
Supplement: Technical Appendix — Supplementary methods for study of spread of a meropenem-resistant Streptococcus pneumoniae serotype 15A-ST63 clone in Japan, 2012–2014. [file 17-1268-Techapp-s1.pdf]

Article DOI: http://dx.doi.org/10.3201/eid2402.171268

Spread of Meropenem-Resistant *Streptococcus pneumoniae* Serotype 15A-ST63 Clone in Japan, 2012–2014

Technical Appendix

Supplementary Methods

We used a core genome single-nucleotide polymorphism (SNP)-based approach to create a phylogenetic tree using the current standard procedure (*1*). To perform this approach, we used Genealogies Unbiased By recomBinations In Nucleotide Sequences (Gubbins) (*2*), which identifies recombination events using an algorithm that iteratively identifies loci containing elevated densities of base substitutions while concurrently constructing a phylogeny based on the putative point mutations outside of these regions. To create input files for Gubbins, we performed raw read mapping followed by duplicate read removal, indel removal, and realignment (*3*).

Core Genome Analysis using Gubbins

Reads from 67 isolates sequenced in this study and reads from 86 global isolates downloaded from Sequence Read Archive (SRA) database (http://www.ncbi.nlm.nih.gov/sra/) underwent quality trimming using Trimmomatic (*4*). Trimmed reads were aligned against a reference genome of *Streptococcus pneumoniae* G54 (NCBI Reference Sequence: NC_011072.11) using the Burrows-Wheeler Aligner (*5*). After the removal of duplicate reads and indels using the GATK Best Practices workflow (*6*), consensus sequences fasta files were created using VCFtools (*7*). Gubbins was run with standard parameters. We created a total of three phylogenic trees using Gubbins. First, we created a tree using all of the isolates (all of the 67 isolates sequenced in this study and 86 global serotype 15A isolates) and *Streptococcus pneumoniae* G54 without any outgroup to find the ancestral strain of the Japanese meropenem-non-susceptible serotype 15A-ST63 strain. We found that there was no candidate for the ancestor

among isolates of any serotype except for 15A. We then created a second phylogenic tree using 35 serotype 15A isolates sequenced in this study and 86 global serotype 15A isolates using *Streptococcus pneumoniae* G54 as an outgroup. This tree generated a clade that included all 24 Japanese meropenem-non-susceptible serotype 15A isolates and six Japanese meropenem-susceptible serotype 15A isolates. Finally, we created a phylogenic tree using these 30 serotype 15A isolates with the PMEN15A-25 isolate used as the outgroup.

Identification of SNPs Specific to Clade-I-MNS

To identify the core genome changes that separated clade-I-MNS from the rest of clade-I, we extracted the core genomes of all of the clade-I isolates and searched for SNPs in Japanese MEPM-NS isolates that were identified in all of the clade-I-MNS isolates and not identified in any of the rest of clade-I. We obtained the core genomes using GET_HOMOLOGS (*8*) and aligned the clustered genes. Then, we identified the SNPs manually. We obtained a total of 1869 core genomes and 550,762 substrates of amino-acid sequences. These SNPs were distributed in 52 genes that are listed in Technical Appendix Table 4.

Genome Assembly

Trimmed reads sequenced in this study were assembled using SPAdes (*9*) with k-mer values ranging from 29 to 101 and in careful mode. Trimmed reads from the downloaded global isolates were assembled using SPAdes with standard parameters and in careful mode. The quality of the assemblies was evaluated using QUAST (*10*).

Comparative Genome Analysis

To define the presence of genes and their alleles, we extracted the target gene regions from the assembled contigs using BLAST+ (*11*). With regard to *pbp1a*, *2b*, and *2x*, we used the corresponding gene sequences from *Streptococcus pneumoniae* G54 as reference sequences (NCBI Reference Sequence: NC_011072.11). The reference sequences used to identify *mefA* (*12*), *mefE* (*13*), *folA* (*14*), *folP* (*14*), *tetO* (*14*), *tetM* (*14*), PI-1 (*rrgA-1*) (*14*), and PI-2 (*pitB-1*) (*14*) are listed in Technical Appendix Table 5.

Estimation of the Date when Meropenem-Non-Susceptible Serotype 15A-ST63 Originated

The result of core genome analysis using Gubbins indicated that the Japanese meropenem-non-susceptible (MEPM-NS) serotype 15A-ST63 strain was derived from the Japanese meropenem-susceptible (MEPM-S) strain. We estimated the date of the most recent common ancestor (MRCA) of each of the two groups using BEAST (*15*). The program was used to analyze the final maximum likelihood tree, the topology of which was fixed, and the alignment of base substitutions occurring outside of putative recombination events using a strict clock model. The ages of the isolates (month and year) were used as input data. Exponential growth was used as the tree prior. The length of chain value was set so that all output values had an effective sample size greater than 200. The analysis estimated that the lineage originated around 1970 (95% credibility interval 1672–2006); the small number of tested isolates may explain the broad credibility interval. In addition, the tree generated in this analysis was slightly different from that in core genome analysis using Gubbins. In this analysis, MEPM-NS isolates were divided into two clades even though MEPM-NS and –S isolates were clearly separated (Technical Appendix Figure 4).

References

1. Robinson ER, Walker TM, Pallen MJ. Genomics and outbreak investigation: from sequence to consequence. Genome Med. 2013;5:36. PubMed http://dx.doi.org/10.1186/gm440

2. Croucher NJ, Page AJ, Connor TR, Delaney AJ, Keane JA, Bentley SD, et al. Rapid phylogenetic analysis of large samples of recombinant bacterial whole genome sequences using Gubbins. Nucleic Acids Res. 2015;43:e15. PubMed http://dx.doi.org/10.1093/nar/gku1196

3. Olson ND, Lund SP, Colman RE, Foster JT, Sahl JW, Schupp JM, et al. Best practices for evaluating single nucleotide variant calling methods for microbial genomics. Front Genet. 2015;6:235. PubMed http://dx.doi.org/10.3389/fgene.2015.00235

4. Bolger AM, Lohse M, Usadel B. Trimmomatic: a flexible trimmer for Illumina sequence data. Bioinformatics. 2014;30:2114–20. PubMed http://dx.doi.org/10.1093/bioinformatics/btu170

5. Li H, Durbin R. Fast and accurate short read alignment with Burrows-Wheeler transform. Bioinformatics. 2009;25:1754–60. PubMed http://dx.doi.org/10.1093/bioinformatics/btp324

6. McKenna A, Hanna M, Banks E, Sivachenko A, Cibulskis K, Kernytsky A, et al. The Genome Analysis Toolkit: a MapReduce framework for analyzing next-generation DNA sequencing data. Genome Res. 2010;20:1297–303. PubMed http://dx.doi.org/10.1101/gr.107524.110

7. Danecek P, Auton A, Abecasis G, Albers CA, Banks E, DePristo MA, et al.; 1000 Genomes Project Analysis Group. The variant call format and VCFtools. Bioinformatics. 2011;27:2156–8. PubMed http://dx.doi.org/10.1093/bioinformatics/btr330

8. Contreras-Moreira B, Vinuesa P. GET_HOMOLOGUES, a versatile software package for scalable and robust microbial pangenome analysis. Appl Environ Microbiol. 2013;79:7696–701. PubMed http://dx.doi.org/10.1128/AEM.02411-13

9. Bankevich A, Nurk S, Antipov D, Gurevich AA, Dvorkin M, Kulikov AS, et al. SPAdes: a new genome assembly algorithm and its applications to single-cell sequencing. J Comput Biol. 2012;19:455–77. PubMed http://dx.doi.org/10.1089/cmb.2012.0021

10. Gurevich A, Saveliev V, Vyahhi N, Tesler G. QUAST: quality assessment tool for genome assemblies. Bioinformatics. 2013;29:1072–5. PubMed http://dx.doi.org/10.1093/bioinformatics/btt086

11. Altschul SF, Gish W, Miller W, Myers EW, Lipman DJ. Basic local alignment search tool. J Mol Biol. 1990;215:403–10. PubMed http://dx.doi.org/10.1016/S0022-2836(05)80360-2

12. Clancy J, Petitpas J, Dib-Hajj F, Yuan W, Cronan M, Kamath AV, et al. Molecular cloning and functional analysis of a novel macrolide-resistance determinant, mefA, from Streptococcus pyogenes. Mol Microbiol. 1996;22:867–79. PubMed http://dx.doi.org/10.1046/j.1365-2958.1996.01521.x

13. Tait-Kamradt A, Clancy J, Cronan M, Dib-Hajj F, Wondrack L, Yuan W, et al. mefE is necessary for the erythromycin-resistant M phenotype in Streptococcus pneumoniae. Antimicrob Agents Chemother. 1997;41:2251–5. PubMed

14. Metcalf BJ, Gertz RE Jr, Gladstone RA, Walker H, Sherwood LK, Jackson D, et al. Strain features and distributions in pneumococci from children with invasive disease before and after 13 valent conjugate vaccine implementation in the United States. Clin Microbiol Infect. 2015. PubMed

15. Drummond AJ, Rambaut A. BEAST: Bayesian evolutionary analysis by sampling trees. BMC Evol Biol. 2007;7:214. PubMed http://dx.doi.org/10.1186/1471-2148-7-214

Technical Appendix Table 1. Strain information and penicillin binding protein profile

Isolate name	Accession no.	Serotype	ST*	Year	Region†	MIC (mg/L) ‡				
						PCG	CTX	MEPM	EM	*pbp1a*:*pbp2b*: *pbp2x*
USA15A-16	ERR065297	15A	63	2004	USA	0.03	NA	NA	0.5	24:27:28
USA15A-10	ERR065320	15A	63	2004	USA	0.12	NA	NA	0.5	24:27:28
USA15A-5	ERR065332	15A	63	2004	USA	0.12	NA	NA	0.5	24:27:28
USA15A-13	ERR068026	15A	63	2004	USA	0.25	NA	NA	0.5	24:27:28
USA15A-14	ERR068028	15A	63	2004	USA	0.25	NA	NA	0.5	24:27:28
USA15A-17	ERR068032	15A	63	2004	USA	0.25	NA	NA	0.5	24:27:28
USA15A-11	ERR068049	15A	63	2004	USA	0.25	NA	NA	0.5	24:27:28
USA15A-18	ERR069724	15A	63	2004	USA	0.25	NA	NA	0.5	24:27:28
USA15A-12	ERR069725	15A	63	2004	USA	0.25	NA	NA	0.5	24:27:28
USA15A-21	ERR124239	15A	63	2007	USA	0.38	NA	NA	256	24:27:28
USA15A-19	ERR124249	15A	63	2007	USA	0.19	NA	NA	256	24:27:28
USA15A-9	ERR124283	15A	63	2007	USA	0.25	NA	NA	256	24:27:28
USA15A-20	ERR124300	15A	63	2007	USA	0.25	NA	NA	32	24:27:28
USA15A-6	ERR129026	15A	63	2007	USA	0.19	NA	NA	256	24:27:28
USA15A-8	ERR129060	15A	63	2007	USA	0.064	NA	NA	256	24:27:28
USA15A-15	ERR129061	15A	63	2007	USA	0.125	NA	NA	256	24:27:28
USA15A-7	ERR129198	15A	63	2007	USA	0.38	NA	NA	256	24:27:28
UK15A-1	ERR1439011	15A	63	2013	UK	NA	NA	NA	NA	24:27:28
UK15A-2	ERR1439047	15A	63	2013	UK	NA	NA	NA	NA	24:27:28
UK15A-3	ERR1439048	15A	63	2013	UK	NA	NA	NA	NA	67:27:35
UK15A-4	ERR1439052	15A	63	2013	UK	NA	NA	NA	NA	24:27:43
UK15A-5	ERR1439054	15A	63	2013	UK	NA	NA	NA	NA	24:27:28
UK15A-6	ERR1439056	15A	63	2013	UK	NA	NA	NA	NA	24:27:28
UK15A-7	ERR1439057	15A	63	2013	UK	NA	NA	NA	NA	24:27:179
UK15A-8	ERR1439069	15A	63	2013	UK	NA	NA	NA	NA	24:27:43
UK15A-9	ERR1439074	15A	63	2013	UK	NA	NA	NA	NA	24:27:179
UK15A-10	ERR1439082	15A	63	2013	UK	NA	NA	NA	NA	67:new3:35
UK15A-11	ERR1439083	15A	63	2013	UK	NA	NA	NA	NA	24:27:8
UK15A-12	ERR1439097	15A	63	2013	UK	NA	NA	NA	NA	24:27:28
UK15A-13	ERR1439101	15A	63	2013	UK	NA	NA	NA	NA	24:27:28
UK15A-14	ERR1439103	15A	63	2013	UK	NA	NA	NA	NA	24:27:179
UK15A-15	ERR1439104	15A	63	2013	UK	NA	NA	NA	NA	24:27:179
UK15A-16	ERR1439112	15A	63	2013	UK	NA	NA	NA	NA	24:27:43
UK15A-17	ERR1439117	15A	63	2013	UK	NA	NA	NA	NA	24:27:28
UK15A-18	ERR1439120	15A	63	2014	UK	NA	NA	NA	NA	24:27:28
UK15A-19	ERR1439131	15A	63	2014	UK	NA	NA	NA	NA	24:27:28
UK15A-20	ERR1439141	15A	63	2014	UK	NA	NA	NA	NA	24:27:28
UK15A-21	ERR1439151	15A	63	2014	UK	NA	NA	NA	NA	new4:27:28
UK15A-22	ERR1439155	15A	63	2014	UK	NA	NA	NA	NA	24:27:28
UK15A-23	ERR1439162	15A	63	2014	UK	NA	NA	NA	NA	24:27:28
UK15A-24	ERR1439167	15A	63	2014	UK	NA	NA	NA	NA	24:27:179
UK15A-25	ERR1439172	15A	63	2014	UK	NA	NA	NA	NA	24:27:28
UK15A-26	ERR1439190	15A	63	2014	UK	NA	NA	NA	NA	67:27:35
UK15A-27	ERR1439193	15A	63	2014	UK	NA	NA	NA	NA	24:27:28
UK15A-28	ERR1439207	15A	63	2014	UK	NA	NA	NA	NA	24:27:28
UK15A-29	ERR1439211	15A	63	2014	UK	NA	NA	NA	NA	24:new4:28
UK15A-30	ERR1439215	15A	63	2014	UK	NA	NA	NA	NA	24:27:28

Isolate name	Accession no.	Serotype	ST*	Year	Region†	MIC (mg/L) ‡ PCG	CTX	MEPM	EM	*pbp1a*:*pbp2b*: *pbp2x*
UK15A-31	ERR1439225	15A	63	2014	UK	NA	NA	NA	NA	24:27:28
UK15A-32	ERR1439240	15A	63	2014	UK	NA	NA	NA	NA	24:27:179
UK15A-33	ERR1439256	15A	63	2014	UK	NA	NA	NA	NA	24:27:28
UK15A-34	ERR1439260	15A	63	2014	UK	NA	NA	NA	NA	24:27:28
UK15A-35	ERR1439264	15A	63	2014	UK	NA	NA	NA	NA	24:27:28
UK15A-36	ERR1439269	15A	63	2014	UK	NA	NA	NA	NA	24:27:179
UK15A-37	ERR1439272	15A	63	2014	UK	NA	NA	NA	NA	24:27:179
UK15A-38	ERR1439276	15A	63	2014	UK	NA	NA	NA	NA	24:27:179
UK15A-39	ERR1439301	15A	63	2014	UK	NA	NA	NA	NA	24:27:28
UK15A-40	ERR1439303	15A	63	2014	UK	NA	NA	NA	NA	67:27:35
UK15A-41	ERR1439306	15A	63	2014	UK	NA	NA	NA	NA	24:27:179
UK15A-42	ERR1439311	15A	63	2014	UK	NA	NA	NA	NA	67:27:35
UK15A-43	ERR1439312	15A	63	2014	UK	NA	NA	NA	NA	24:27:28
UK15A-44	ERR1439320	15A	63	2014	UK	NA	NA	NA	NA	new5:27:28
UK15A-45	ERR1439325	15A	63	2014	UK	NA	NA	NA	NA	new5:27:28
UK15A-46	ERR1439332	15A	63	2014	UK	NA	NA	NA	NA	24:27:179
UK15A-47	ERR1439335	15A	63	2014	UK	NA	NA	NA	NA	67:27:35
UK15A-48	ERR1439338	15A	63	2014	UK	NA	NA	NA	NA	24:27:28
UK15A-49	ERR1439348	15A	63	2014	UK	NA	NA	NA	NA	67:27:35
UK15A-50	ERR1439365	15A	63	2014	UK	NA	NA	NA	NA	67:27:35
UK15A-51	ERR1439384	15A	63	2014	UK	NA	NA	NA	NA	24:27:179
UK15A-52	ERR1439400	15A	63	2014	UK	NA	NA	NA	NA	67:27:35
UK15A-53	ERR1439403	15A	63	2014	UK	NA	NA	NA	NA	67:27:35
UK15A-54	ERR1439412	15A	63	2014	UK	NA	NA	NA	NA	24:27:28
UK15A-55	ERR1439442	15A	63	2014	UK	NA	NA	NA	NA	24:27:28
UK15A-56	ERR1439469	15A	63	2014	UK	NA	NA	NA	NA	24:27:28
UK15A-57	ERR1439551	15A	63	2014	UK	NA	NA	NA	NA	67:27:35
UK15A-58	ERR1439560	15A	63	2014	UK	NA	NA	NA	NA	24:27:28
USA15A-1	ERR505735	15A	63	NA	USA	NA	NA	NA	NA	24:27:35
USA15A-2	ERR600092	15A	63	NA	USA	NA	NA	NA	NA	24:27:28
USA15A-3	ERR600173	15A	63	NA	USA	NA	NA	NA	NA	24:27:28
USA15A-4	ERR600180	15A	63	NA	USA	NA	NA	NA	NA	24:73:114
MR15A-2_PC118	DRR098620	15A	63	2012	JP(Chiba)	4	0.5	0.5	>128	13:new1:43
MR15A-3_PC126	DRR098621	15A	63	2012	JP(Osaka)	2	1	0.5	>128	13:new1:43
MR15A-1_PC13	DRR098624	15A	63	2012	JP(Chiba)	2	0.5	0.5	>128	13:new1:43
MR15A-4_PC131	DRR098623	15A	63	2012	JP(Chiba)	2	0.5	0.5	>128	13:new1:43
MS15A-6_PC283	DRR098637	15A	63	2013	JP(Hokkaido)	0.25	≦0.06	≦0.06	>128	24:27:28
MR15A-5_PC206	DRR098628	15A	63	2013	JP(Yamaguchi)	4	1	0.5	>128	13:new1:43
MS15A-8_PC358	DRR098648	15A	63	2013	JP(Hokkaido)	0.25	0.12	≦0.06	>128	24:27:28
MS15A-11_PC723	DRR098683	15A	63	2014	JP(Yamagata)	0.25	≦0.06	≦0.06	>128	24:27:28
MS15A-1_PC16	DRR098626	15A	63	2012	JP(Kyoto)	0.25	0.5	≦0.06	>128	24:27:43
MR15A-6_PC282	DRR098636	15A	63	2013	JP(Saga)	2	0.5	0.5	>128	13:new1:43
MS15A-4_PC239	DRR098632	15A	63	2013	JP(Gifu)	0.25	0.5	≦0.06	>128	24:27:43
MS15A-5_PC273	DRR098635	15A	63	2012	JP(Okayama)	0.25	0.25	≦0.06	>128	24:27:43
MR15A-20_PC313	DRR098642	15A	63	2013	JP(Yamaguchi)	4	4	1	>128	13:new1:new3
MR15A-7_PC324	DRR098643	15A	63	2013	JP(Shizuoka)	2	0.5	0.5	>128	13:new1:43
MR15A-21_PC342	DRR098645	15A	63	2013	JP(Osaka)	2	1	1	>128	13:new1:43
MS15A-7_PC307	DRR098641	15A	63	2013	JP(Tokyo)	0.5	0.5	≦0.06	>128	24:27:43

Isolate name	Accession no.	Serotype	ST*	Year	Region†	MIC (mg/L) ‡				*pbp1a*:*pbp2b*: *pbp2x*
						PCG	CTX	MEPM	EM	
MR15A-8_PC376	DRR098649	15A	63	2013	JP(Tokushima)	2	0.5	0.5	>128	13:new1:43
MR15A-9_PC418	DRR098654	15A	63	2013	JP(Okayama)	2	0.5	0.5	>128	13:new1:43
MR15A-10_PC441	DRR098655	15A	63	2013	JP(Yamaguchi)	2	0.5	0.5	>128	13:new1:43
MS15A-9_PC443	DRR098656	15A	63	2013	JP(Hokkaido)	0.25	0.25	≦0.06	>128	24:27:43
MR15A-11_PC495	DRR098659	15A	63	2013	JP(Osaka)	2	0.5	0.5	>128	13:new1:43
MR15A-22_PC518	DRR098663	15A	63	2014	JP(Yamaguchi)	4	1	1	>128	13:new1:43
MR15A-12_PC572	DRR098666	15A	63	2014	JP(Gifu)	2	0.5	0.5	>128	13:new1:43
MR15A-13_PC618	DRR098670	15A	63	2014	JP(Saga)	2	0.5	0.5	>128	13:new1:43
MR15A-14_PC620	DRR098671	15A	63	2014	JP(Chiba)	1	0.5	0.5	>128	13:new1:43
MR15A-23_PC624	DRR098672	15A	63	2014	JP(Chiba)	2	0.5	1	>128	13:new1:43
MR15A-15_PC638	DRR098673	15A	63	2014	JP(Wakayama)	2	0.5	0.5	>128	13:new1:43
MR15A-16_PC646	DRR098676	15A	63	2014	JP(Ibaraki)	1	0.25	0.5	>128	13:new1:43
MR15A-17_PC654	DRR098677	15A	63	2013	JP(Yamaguchi)	2	4	0.5	>128	13:new1:new6
MR15A-24_PC662	DRR098678	15A	63	2014	JP(Ohita)	2	0.5	1	>128	13:new1:43
MR15A-18_PC686	DRR098680	15A	63	2014	JP(Wakayama)	1	0.5	0.5	>128	13:new1:43
MS15A-3_PC228	DRR098631	15A	63	2013	JP(Yamagata)	0.5	1	≦0.06	>128	24:27:112
MR15A-19_PC718	DRR098682	15A	63	2014	JP(Kanagawa)	2	0.5	0.5	>128	13:new1:43
MS15A-10_PC702	DRR098681	15A	63	2014	JP(Miyagi)	0.25	0.25	≦0.06	>128	24:27:new1
MR15BC-2_PC267	DRR098634	15B/C	83	2012	JP(Okayama)	2	1	0.5	>128	15:12:18
MR15BC-3_PC516	DRR098662	15B/C	83	2014	JP(Kumamoto)	2	1	1	>128	15:12:18
MR15BC-1_PC227	DRR098630	15B/C	3934	2013	JP(Kyoto)	1	1	0.5	>128	new1:31:new2
MR19A-8_PC297	DRR098639	19A	320	2013	JP(Kumamoto)	4	2	1	>128	13:11:16
MR19A-9_PC396	DRR098652	19A	320	2013	JP(Gifu)	4	1	1	>128	13:11:16
MR19A-10_PC576	DRR098668	19A	320	2014	JP(Osaka)	4	2	1	>128	13:11:16
MR19A-11_PC641	DRR098675	19A	320	2014	JP(Miyazaki)	4	2	1	>128	13:11:16
MR19A-2_PC345	DRR098646	19A	3111	2013	JP(Yamaguchi)	2	1	0.5	>128	13:24:112
MR19A-3_PC381	DRR098650	19A	3111	2013	JP(Saga)	1	0.5	0.5	>128	13:24:112
MR19A-4_PC475	DRR098658	19A	3111	2014	JP(Kanagawa)	2	1	0.5	>128	13:24:112
MR19A-5_PC505	DRR098661	19A	3111	2014	JP(Yamaguchi)	2	1	0.5	>128	13:24:112
MR19A-6_PC543	DRR098665	19A	3111	2014	JP(Gifu)	1	1	0.5	>128	13:24:112
MR19A-7_PC583	DRR098669	19A	3111	2014	JP(Yamaguchi)	1	0.5	0.5	>128	13:24:112
MR19A-1_PC93	DRR098685	19A	3111	2012	JP(Tokyo)	1	1	0.5	>128	new2:16:112
MR19F-2_PC306	DRR098640	19F	115	2013	JP(Shizuoka)	2	1	0.5	16	13:31:47
MR19F-3_PC463	DRR098657	19F	236	2013	JP(Shizuoka)	2	1	0.5	4	13:7:8
MR19F-1_PC49	DRR098660	19F	236	2012	JP(Yamaguchi)	2	1	0.5	4	13:16:47
MR23F-1_PC329	DRR098644	23F	242	2013	JP(Kanagawa)	2	1	0.5	>128	13:31:73
MR35B-1_PC129	DRR098622	35B	558	2012	JP(Chiba)	2	1	0.5	>128	4:7:7
MR35B-2_PC216	DRR098629	35B	558	2013	JP(Yamaguchi)	1	1	0.5	16	4:7:7
MR35B-3_PC291	DRR098638	35B	558	2013	JP(Yamaguchi)	1	1	0.5	2	4:7:7
MR35B-4_PC357	DRR098647	35B	558	2013	JP(Ohita)	1	0.5	0.5	4	4:7:7
MR35B-5_PC393	DRR098651	35B	558	2013	JP(Osaka)	1	0.5	0.5	≦0.06	4:7:7
MR35B-8_PC540	DRR098664	35B	558	2014	JP(Yamaguchi)	2	1	1	8	4:7:7
MR35B-6_PC574	DRR098667	35B	558	2014	JP(Yamaguchi)	1	0.5	0.5	8	4:7:7
MR35B-7_PC640	DRR098674	35B	558	2014	JP(Yamaguchi)	2	1	0.5	0.12	4:7:7
MR6A-1_PC240	DRR098633	6A	2756	2013	JP(Chiba)	2	1	0.5	>128	13:31:73
MR6B-1_PC140	DRR098625	6B	9335	2013	JP(Kumamoto)	4	4	2	>128	13:49:new7
MR6D-1_PC80	DRR098684	6D	282	2012	JP(Gifu)	2	1	0.5	4	15:12:18
MRUT-3_PC676	DRR098679	NT	4845	2014	JP(Chiba)	2	1	0.5	4	13:16:new4

Isolate name	Accession no.	Serotype	ST*	Year	Region†	MIC (mg/L) ‡ PCG	CTX	MEPM	EM	*pbp1a*:*pbp2b*: *pbp2x*
MRUT-1_PC192	DRR098627	NT	7502	2013	JP(Chiba)	2	1	0.5	>128	new3:16:new5
MRUT-2_PC400	DRR098653	NT	10253	2013	JP(Chiba)	2	1	1	16	13:new2:new4
PMEN15A-25	DRR098686	15A	63	1998	PTG	0.064	0.047	NA	>256	24:27:28
CN15A-1	SRR3211689	15A	63	2012	CN	NA	NA	NA	NA	24:27:28
CN15A-2	SRR3211690	15A	63	2011	CN	NA	NA	NA	NA	24:27:28
CN15A-3	SRR3211691	15A	63	2010	CN	NA	NA	NA	NA	24:27:28
CN15A-4	SRR3211692	15A	63	2010	CN	NA	NA	NA	NA	24:27:8
CN15A-5	SRR3211693	15A	63	2009	CN	NA	NA	NA	NA	24:27:28
CN15A-6	SRR3211694	15A	63	2013	CN	NA	NA	NA	NA	24:27:11
CN15A-7	SRR3211695	15A	63	2013	CN	NA	NA	NA	NA	24:27:28

*ST, sequence type.
†JP, Japan; PTG, Portugal; CN, Canada.
‡MIC, MIC; PCG, penicillin G; CTX, cefotaxime; MEPM, meropenem.

Technical Appendix Table 2. Mapping and assembly statistics

Isolate name	Mapping to the *Streptococcus pneumoniae* G54 genome: Depth of coverage	Breadth of coverage	No. of contigs	N50	Length of the longest contig	No. of bases in contigs	No. of contigs >1K	No. of bases in contigs >1K
USA15A-16	304	99.2	141	51192	103936	2048435	73	2042765
USA15A-10	191	99.2	160	47739	233907	2091039	71	2083988
USA15A-5	232	99.2	138	47835	143850	2048562	73	2041473
USA15A-13	282	98.5	144	53272	117879	2035279	72	2029302
USA15A-14	325	98.4	153	56296	152786	2032756	74	2027545
USA15A-17	300	99.2	155	56559	233907	2091321	69	2082546
USA15A-11	260	99.2	146	45009	233947	2093768	76	2085899
USA15A-18	348	99.2	140	51042	144023	2048081	74	2041778
USA15A-12	344	99.2	135	47739	144057	2049657	73	2044263
USA15A-21	560	99.3	162	42946	143978	2043675	79	2037099
USA15A-19	408	99.3	135	44285	117742	2046667	77	2038963
USA15A-9	347	99.3	143	44792	152913	2046100	79	2040668
USA15A-20	672	99.2	147	51192	187683	2042774	75	2036720
USA15A-6	248	98.6	162	44792	152699	2099233	83	2090928
USA15A-8	220	99.2	168	53272	233922	2087910	74	2079149
USA15A-15	179	99.2	159	45009	233754	2089415	73	2082391
USA15A-7	222	98.5	132	47085	117879	2031971	74	2026455
UK15A-1	132	99.3	214	58499	229361	2037703	68	2031997
UK15A-2	182	99.3	160	62277	197139	2048111	67	2044751
UK15A-3	119	99.1	195	48666	143748	2044407	75	2038369
UK15A-4	126	99.3	191	53272	176414	2084663	74	2073611
UK15A-5	151	99.3	248	53272	193632	2077034	72	2069614
UK15A-6	103	99.3	187	54688	192035	2073297	74	2067279
UK15A-7	155	99.3	226	48060	178057	2048686	78	2042765
UK15A-8	149	99.3	241	51192	225219	2116554	69	2109936
UK15A-9	88	99.2	178	47835	118013	2071299	79	2065697
UK15A-10	253	99.0	182	53271	118454	2044161	69	2042157
UK15A-11	118	98.6	196	56406	180443	2065809	72	2060528

Isolate name	Mapping to the *Streptococcus pneumoniae* G54 genome: Depth of coverage	Breadth of coverage	No. of contigs	N50	Length of the longest contig	No. of bases in contigs	No. of contigs >1K	No. of bases in contigs >1K
UK15A-12	200	99.3	173	58499	225347	2039728	66	2034520
UK15A-13	77	99.3	206	51192	156502	2079916	75	2071096
UK15A-14	146	99.3	232	50391	178296	2051027	78	2044565
UK15A-15	99	99.3	171	53856	178235	2050989	70	2046995
UK15A-16	100	99.3	186	51192	176297	2079776	71	2072361
UK15A-17	125	99.3	262	53271	193632	2079982	71	2070191
UK15A-18	123	99.3	246	61081	225434	2082353	72	2070637
UK15A-19	117	98.8	192	58499	117503	2035192	67	2030023
UK15A-20	89	99.1	200	51192	176387	2041622	71	2036381
UK15A-21	117	99.3	204	60794	225263	2077499	66	2068802
UK15A-22	150	99.4	240	58444	228921	2077747	68	2068774
UK15A-23	106	98.6	193	57846	236394	2042302	64	2038575
UK15A-24	133	99.3	211	53634	118472	2052430	75	2047401
UK15A-25	97	99.3	164	63724	176634	2047916	69	2044551
UK15A-26	133	99.3	189	56827	143871	2049031	76	2045888
UK15A-27	92	99.3	185	58499	193731	2036346	67	2031070
UK15A-28	89	99.3	233	57830	193867	2051939	71	2045227
UK15A-29	117	99.3	205	51068	176372	2054460	76	2050083
UK15A-30	136	98.8	220	51192	118294	2081000	80	2075119
UK15A-31	204	99.3	273	51116	176371	2075659	82	2070386
UK15A-32	96	99.2	151	53856	177486	2051253	75	2044746
UK15A-33	131	99.1	235	56192	193632	2048628	66	2041010
UK15A-34	118	99.3	212	49154	143224	2081720	71	2072929
UK15A-35	127	99.3	209	55844	193497	2078248	71	2071923
UK15A-36	154	99.3	261	46979	178092	2055562	83	2045448
UK15A-37	122	99.3	255	53272	177499	2045811	79	2038719
UK15A-38	217	99.3	234	51448	177962	2050295	80	2044954
UK15A-39	144	99.3	285	53272	159120	2077351	76	2069143
UK15A-40	110	99.1	169	57310	143842	2049091	72	2045941
UK15A-41	145	99.3	191	53272	109932	2051289	75	2047008
UK15A-42	140	99.1	202	53272	143917	2047090	75	2043437
UK15A-43	124	99.3	245	43693	176416	2076837	78	2070426
UK15A-44	111	99.3	235	55868	193632	2077733	69	2066489
UK15A-45	112	99.3	207	58455	225479	2077621	70	2071045
UK15A-46	114	99.3	167	48430	118059	2054430	74	2048233
UK15A-47	121	98.4	197	56827	146082	2035926	66	2032991
UK15A-48	111	99.3	197	56706	221207	2079591	64	2071838
UK15A-49	55	99.1	175	56854	153042	2047339	68	2041772
UK15A-50	51	99.1	202	56853	152737	2046932	64	2043140
UK15A-51	130	99.3	249	50897	123903	2049366	76	2042712
UK15A-52	56	99.1	184	56523	152583	2045056	68	2041360
UK15A-53	76	99.1	157	56310	117880	2049111	66	2044879
UK15A-54	77	99.2	361	51030	132164	2110478	80	2085286
UK15A-55	120	99.3	171	58499	193632	2048872	66	2044600
UK15A-56	131	99.1	199	61010	118168	2041390	69	2037074
UK15A-57	70	99.1	207	51192	143883	2047636	73	2042891
UK15A-58	102	99.3	200	57876	212581	2079069	67	2070199
USA15A-1	180	99.2	152	49187	131386	2117994	77	2113421

Isolate name	Mapping to the *Streptococcus pneumoniae* G54 genome: Depth of coverage	Breadth of coverage	No. of contigs	N50	Length of the longest contig	No. of bases in contigs	No. of contigs >1K	No. of bases in contigs >1K
USA15A-2	359	99.2	129	57876	144296	2051367	69	2048072
USA15A-3	211	99.2	135	53272	116057	2090483	70	2086286
USA15A-4	226	99.2	148	46449	147861	2101459	74	2095036
MR15A-2_PC118	59	99.2	123	62362	236423	2087922	58	2077854
MR15A-3_PC126	48	99.2	107	74297	196965	2088018	57	2084746
MR15A-1_PC13	55	99.2	113	74297	187507	2089105	60	2086082
MR15A-4_PC131	37	99.2	115	73668	237180	2091099	58	2086559
MS15A-6_PC283	60	98.9	73	88601	205515	2055844	42	2054130
MR15A-5_PC206	51	99.2	86	76986	281100	2091251	48	2088027
MS15A-8_PC358	47	98.9	69	76464	205483	2054767	48	2053053
MS15A-11_PC723	60	98.9	78	76981	230301	2056696	49	2053450
MS15A-1_PC16	57	99.2	118	73668	187380	2091927	59	2083695
MR15A-6_PC282	53	99.2	113	74102	273917	2092055	51	2085832
MS15A-4_PC239	52	99.3	161	57166	236577	2125628	66	2115140
MS15A-5_PC273	53	99.2	110	87855	236295	2090064	49	2086830
MR15A-20_PC313	37	99.2	112	67201	144493	2092043	59	2087708
MR15A-7_PC324	69	99.2	109	74198	281111	2088707	58	2084579
MR15A-21_PC342	46	99.2	116	57166	187366	2089651	62	2083161
MS15A-7_PC307	69	99.2	91	74131	281906	2090717	47	2086830
MR15A-8_PC376	53	99.2	107	68791	281098	2084758	57	2079883
MR15A-9_PC418	62	99.2	101	73034	280619	2088689	52	2083075
MR15A-10_PC441	64	99.0	90	73668	207867	2087893	54	2082810
MS15A-9_PC443	51	98.9	110	73704	188362	2085036	55	2078197
MR15A-11_PC495	48	99.2	102	89777	200421	2132327	51	2126700
MR15A-22_PC518	46	99.2	100	62808	236982	2130579	66	2125771
MR15A-12_PC572	39	99.2	130	76941	208418	2089200	61	2083124
MR15A-13_PC618	48	99.1	103	63383	230465	2090072	66	2083524
MR15A-14_PC620	53	99.2	106	69164	272921	2090665	53	2083387
MR15A-23_PC624	46	99.1	136	51524	166238	2091029	71	2083783
MR15A-15_PC638	66	99.1	91	68716	279775	2091648	51	2087003
MR15A-16_PC646	63	99.1	92	69042	228810	2090923	54	2085392
MR15A-17_PC654	59	99.2	94	67201	235803	2087351	52	2080862
MR15A-24_PC662	40	99.0	128	73952	236444	2075919	55	2070184
MR15A-18_PC686	54	99.2	137	57004	198691	2125849	73	2117678
MS15A-3_PC228	53	99.2	120	67201	236104	2126272	60	2121183
MR15A-19_PC718	53	99.2	105	63747	200061	2087635	58	2081976
MS15A-10_PC702	49	98.9	81	67201	241587	2056026	52	2051754
MR15BC-2_PC267	45	93.5	117	53186	236869	2141903	67	2134165
MR15BC-3_PC516	50	93.3	83	72791	157706	2137629	58	2134657
MR15BC-1_PC227	54	92.7	113	69382	182768	2175295	56	2167627
MR19A-8_PC297	33	91.5	105	66211	199669	2042136	59	2036366
MR19A-9_PC396	39	91.5	87	69312	199865	2039606	52	2035554
MR19A-10_PC576	57	91.6	96	74991	211348	2041305	53	2037811
MR19A-11_PC641	44	91.5	82	68832	211347	2061370	53	2056600
MR19A-2_PC345	47	91.6	120	72932	188518	2117172	56	2112601
MR19A-3_PC381	52	91.6	109	94314	197604	2077760	47	2076395
MR19A-4_PC475	42	91.6	104	67202	197594	2082008	52	2075788
MR19A-5_PC505	52	91.7	106	69709	294446	2080140	53	2076838

Isolate name	Mapping to the *Streptococcus pneumoniae* G54 genome: Depth of coverage	Mapping to the *Streptococcus pneumoniae* G54 genome: Breadth of coverage	No. of contigs	N50	Length of the longest contig	No. of bases in contigs	No. of contigs >1K	No. of bases in contigs >1K
MR19A-6_PC543	21	91.5	138	57261	162486	2075625	75	2069618
MR19A-7_PC583	46	91.6	101	80323	197714	2080190	53	2074623
MR19A-1_PC93	39	91.6	110	64498	196287	2102697	53	2098658
MR19F-2_PC306	50	91.7	96	61356	288923	2060117	55	2055310
MR19F-3_PC463	59	91.6	105	67940	171348	2079289	56	2074660
MR19F-1_PC49	49	91.7	88	66608	289458	2039739	52	2035015
MR23F-1_PC329	45	92.3	137	61061	162764	2102475	61	2096379
MR35B-1_PC129	48	91.5	78	89013	155318	2016531	46	2015165
MR35B-2_PC216	59	91.4	76	88920	145836	2011423	43	2010919
MR35B-3_PC291	62	91.4	58	90869	141297	2012520	39	2011858
MR35B-4_PC357	56	92.6	73	90899	209053	2099214	41	2098690
MR35B-5_PC393	57	90.7	61	72699	154064	1998062	43	1998062
MR35B-8_PC540	36	91.4	164	75362	142333	2011760	49	2006412
MR35B-6_PC574	36	91.3	75	72710	115364	2013350	50	2012442
MR35B-7_PC640	43	90.6	262	45845	93591	2001819	75	1999736
MR6A-1_PC240	24	91.5	104	58856	165469	2028967	69	2020675
MR6B-1_PC140	47	92.8	127	48692	133952	2109566	78	2103163
MR6D-1_PC80	37	93.3	98	74289	130520	2139735	60	2137312
MRUT-3_PC676	39	91.2	359	49208	130039	2043252	81	2036159
MRUT-1_PC192	40	90.8	78	74395	195591	2049166	56	2046931
MRUT-2_PC400	49	90.9	94	81424	194089	2073449	48	2068790
PMEN15A-25	53	99.3	83	87810	282871	2061197	46	2058561
CN15A-1	112	99.2	98	61054	186488	2056469	59	2051810
CN15A-2	254	99.2	103	59932	203611	2054693	57	2051869
CN15A-3	186	99.2	83	73950	292862	2060598	49	2056702
CN15A-4	110	98.6	82	74405	280305	2079105	50	2076726
CN15A-5	148	99.2	85	74079	292589	2060269	48	2056975
CN15A-6	247	99.2	113	63398	241787	2058721	62	2055025
CN15A-7	197	99.2	87	74264	159798	2061429	48	2057766

Technical Appendix Table 3. Antimicrobial resistance genes and pilus determinants

Isolate name	*tetO*	*tetM*	*tetM* stop codon insertion	*ermB*	*ermTR*	*mef*	*folA* mutation	*folP* insertion	*pili1*	*pili2*
USA15A-16	–	+	–	+	–	–	–	–	–	–
USA15A-10	–	+	–	+	–	–	+	+	–	–
USA15A-5	–	+	–	+	–	–	–	+	–	–
USA15A-13	–	+	–	+	–	–	–	+	–	–
USA15A-14	–	+	–	+	–	–	–	+	–	–
USA15A-17	–	+	–	+	–	–	+	+	–	–
USA15A-11	–	+	–	+	–	–	+	+	–	–
USA15A-18	–	+	–	+	–	–	–	–	–	–
USA15A-12	–	+	–	+	–	–	–	–	–	–
USA15A-21	–	+	–	+	–	–	–	–	–	–
USA15A-19	–	+	–	+	–	–	–	–	–	–
USA15A-9	–	+	–	+	–	–	–	–	–	–
USA15A-20	–	+	–	+	–	–	–	–	–	–
USA15A-6	–	+	–	+	–	–	–	+	–	–
USA15A-8	–	+	–	+	–	–	+	+	–	–
USA15A-15	–	+	–	+	–	–	+	+	–	–
USA15A-7	–	+	–	+	–	–	–	+	–	–
UK15A-1	–	+	–	+	–	–	–	–	–	–
UK15A-2	–	+	–	+	–	–	–	–	–	–
UK15A-3	–	+	–	+	–	–	+	+	–	–
UK15A-4	–	+	–	+	–	–	–	–	–	–
UK15A-5	–	+	+	+	–	–	–	–	–	–
UK15A-6	–	+	–	+	–	–	–	–	–	–
UK15A-7	–	+	–	+	–	–	–	–	–	–
UK15A-8	–	+	–	+	–	–	–	–	–	–
UK15A-9	–	+	–	+	–	–	–	–	–	–
UK15A-10	–	+	–	+	–	–	+	+	–	–
UK15A-11	–	+	–	+	–	–	–	–	–	–
UK15A-12	–	+	–	+	–	–	–	–	–	–
UK15A-13	–	+	–	+	–	–	–	–	–	–
UK15A-14	–	+	–	+	–	–	–	–	–	–
UK15A-15	–	+	–	+	–	–	–	–	–	–
UK15A-16	–	+	–	+	–	–	–	–	–	–
UK15A-17	–	+	–	+	–	–	–	–	–	–
UK15A-18	–	+	–	+	–	–	–	–	–	–
UK15A-19	–	+	–	+	–	–	–	–	–	–
UK15A-20	–	+	–	+	–	–	–	–	–	–
UK15A-21	–	+	+	+	–	–	–	–	–	–
UK15A-22	–	+	+	+	–	–	–	–	–	–
UK15A-23	–	+	–	+	–	–	–	–	–	–
UK15A-24	–	+	–	+	–	–	–	–	–	–
UK15A-25	–	+	–	+	–	–	–	–	–	–
UK15A-26	–	+	–	+	–	–	+	+	–	–
UK15A-27	–	+	–	+	–	–	–	–	–	–
UK15A-28	–	+	–	+	–	–	–	–	–	–
UK15A-29	–	+	+	+	–	–	–	–	–	–
UK15A-30	–	+	–	+	–	–	–	–	–	–
UK15A-31	–	+	+	+	–	–	–	–	–	–
UK15A-32	–	+	–	+	–	–	–	–	–	–
UK15A-33	–	+	+	+	–	–	–	–	–	–
UK15A-34	–	+	+	+	–	–	–	–	–	–
UK15A-35	–	+	+	+	–	–	–	–	–	–
UK15A-36	–	+	–	+	–	–	–	–	–	–
UK15A-37	–	+	–	+	–	–	–	–	–	–
UK15A-38	–	+	–	+	–	–	–	–	–	–
UK15A-39	–	+	–	+	–	–	–	–	–	–
UK15A-40	–	+	–	+	–	–	+	+	–	–
UK15A-41	–	+	–	+	–	–	–	–	–	–
UK15A-42	–	+	–	+	–	–	+	+	–	–
UK15A-43	–	+	+	+	–	–	–	–	–	–
UK15A-44	–	+	+	+	–	–	–	–	–	–
UK15A-45	–	+	+	+	–	–	–	–	–	–
UK15A-46	–	+	–	+	–	–	–	–	–	–
UK15A-47	–	+	–	+	–	–	+	+	–	–
UK15A-48	–	+	+	+	–	–	–	–	–	–
UK15A-49	–	+	–	+	–	–	+	+	–	–
UK15A-50	–	+	–	+	–	–	+	+	–	–
UK15A-51	–	+	–	+	–	–	–	–	–	–

Isolate name	*tetO*	*tetM*	*tetM* stop codon insertion	*ermB*	*ermTR*	*mef*	*folA* mutation	*folP* insertion	*pili1*	*pili2*
UK15A-52	–	+	–	+	–	–	+	+	–	–
UK15A-53	–	+	–	+	–	–	+	+	–	–
UK15A-54	–	+	–	+	–	–	–	–	–	–
UK15A-55	–	+	–	+	–	–	–	–	–	–
UK15A-56	–	+	–	+	–	–	–	–	–	–
UK15A-57	–	+	–	+	–	–	+	+	–	–
UK15A-58	–	+	–	+	–	–	–	–	–	–
USA15A-1	–	+	–	+	–	–	–	–	–	–
USA15A-2	–	+	–	+	–	–	–	–	–	–
USA15A-3	–	+	–	+	–	–	–	+	–	–
USA15A-4	–	+	–	+	–	–	–	+	–	–
MR15A-2_PC118	–	+	–	+	–	–	–	–	–	–
MR15A-3_PC126	–	+	–	+	–	–	–	–	–	–
MR15A-1_PC13	–	+	–	+	–	–	–	–	–	–
MR15A-4_PC131	–	+	–	+	–	–	–	–	–	–
MS15A-6_PC283	–	+	–	+	–	–	–	–	–	–
MR15A-5_PC206	–	+	–	+	–	–	–	–	–	–
MS15A-8_PC358	–	+	–	+	–	–	–	–	–	–
MS15A-11_PC723	–	+	–	+	–	–	–	–	–	–
MS15A-1_PC16	–	+	–	+	–	–	–	–	–	–
MR15A–6_PC282	-	+	–	+	–	–	–	–	–	–
MS15A-4_PC239	–	+	–	+	–	–	–	–	–	–
MS15A-5_PC273	–	+	–	+	–	–	–	–	–	–
MR15A-20_PC313	–	+	–	+	–	–	–	–	–	–
MR15A-7_PC324	–	+	–	+	–	–	–	–	–	–
MR15A-21_PC342	–	+	–	+	–	–	–	–	–	–
MS15A-7_PC307	–	+	–	+	–	–	–	–	–	–
MR15A-8_PC376	–	+	–	+	–	–	–	–	–	–
MR15A-9_PC418	–	+	–	+	–	–	–	–	–	–
MR15A-10_PC441	–	+	–	+	–	–	–	–	–	–
MS15A-9_PC443	–	+	–	+	–	–	–	–	–	–
MR15A-11_PC495	–	+	–	+	–	–	–	–	–	–
MR15A-22_PC518	–	+	–	+	–	–	–	–	–	–
MR15A-12_PC572	–	+	–	+	–	–	–	–	–	–
MR15A-13_PC618	–	+	–	+	–	–	–	–	–	–
MR15A-14_PC620	–	+	–	+	–	–	–	–	–	–
MR15A-23_PC624	–	+	–	+	–	–	–	–	–	–
MR15A-15_PC638	–	+	–	+	–	–	–	–	–	–
MR15A-16_PC646	–	+	–	+	–	–	–	–	–	–
MR15A-17_PC654	–	+	–	+	–	–	–	–	–	–
MR15A-24_PC662	–	+	–	+	–	–	–	–	–	–
MR15A-18_PC686	–	+	–	+	–	–	–	–	–	–
MS15A-3_PC228	–	+	–	+	–	–	–	–	–	–
MR15A-19_PC718	–	+	–	+	–	–	–	–	–	–
MS15A-10_PC702	–	+	+	+	–	–	–	–	–	–
MR15BC-2_PC267	–	+	–	+	–	–	+	+	–	–
MR15BC-3_PC516	–	+	–	+	–	–	+	+	–	–
MR15BC-1_PC227	–	+	–	+	–	–	–	–	–	–
MR19A-8_PC297	–	+	–	+	–	E	+	+	+	+
MR19A-9_PC396	–	+	–	+	–	E	+	+	+	+
MR19A-10_PC576	–	+	–	+	–	E	+	+	+	+
MR19A-11_PC641	–	+	–	+	–	E	+	+	+	+
MR19A-2_PC345	–	+	–	+	–	E	–	–	+	–
MR19A-3_PC381	–	+	–	+	–	E	–	–	+	–
MR19A-4_PC475	–	+	–	+	–	E	–	–	+	–
MR19A-5_PC505	–	+	–	+	–	E	–	–	+	–
MR19A-6_PC543	–	+	–	+	–	E	–	–	+	–
MR19A-7_PC583	–	+	–	+	–	E	–	–	+	–
MR19A-1_PC93	–	+	–	+	–	E	–	–	+	–
MR19F-2_PC306	–	+	–	+	–	–	–	+	+	+
MR19F-3_PC463	–	+	–	–	–	E	–	–	+	+
MR19F-1_PC49	–	+	–	–	–	E	–	+	+	+
MR23F-1_PC329	–	+	–	+	–	–	–	+	+	–
MR35B-1_PC129	–	+	–	+	–	E	–	–	+	–
MR35B-2_PC216	–	+	–	–	–	E	–	–	+	–
MR35B-3_PC291	–	+	–	–	–	E	–	–	+	–
MR35B-4_PC357	–	+	–	–	–	E	–	–	+	–
MR35B-5_PC393	–	–	–	–	–	–	–	–	+	–
MR35B-8_PC540	–	+	–	–	–	E	–	–	+	–

Isolate name	*tetO*	*tetM*	*tetM* stop codon insertion	*ermB*	*ermTR*	*mef*	*folA* mutation	*folP* insertion	*pili1*	*pili2*
MR35B-6_PC574	–	+	–	–	–	E	–	–	+	–
MR35B-7_PC640	–	–	–	–	–	–	–	–	+	–
MR6A-1_PC240	–	+	–	+	–	–	–	+	–	–
MR6B-1_PC140	–	+	–	+	–	E	–	+	+	–
MR6D-1_PC80	–	+	–	–	–	E	+	–	–	–
MRUT-3_PC676	–	+	–	–	–	E	–	+	–	–
MRUT-1_PC192	–	+	–	+	–	–	–	+	–	–
MRUT-2_PC400	–	+	–	–	–	E	–	+	–	–
PMEN15A-25	–	+	+	+	–	–	–	–	–	–
CN15A-1	–	+	–	+	–	–	–	–	–	–
CN15A-2	–	+	–	+	–	–	–	–	–	–
CN15A-3	–	+	–	+	–	–	–	–	–	–
CN15A-4	–	+	–	+	–	–	–	–	–	–
CN15A-5	–	+	–	+	–	–	–	–	–	–
CN15A-6	–	+	–	+	–	–	–	+	–	–
CN15A-7	–	+	–	+	–	–	–	–	–	–

Technical Appendix Table 4. Core genome changes that separate clade-I-MNS from the rest of clade I

Gene name	Sequence ID
ABC transporter [Streptococcus pneumoniae]	WP_001814375.1
ABC transporter ATP binding protein [Streptococcus pneumoniae]	SNJ41425.1
ABC transporter ATP binding protein [Streptococcus pneumoniae]	WP_083990072.1
ABC transporter permease [Streptococcus pneumoniae]	WP_054383052.1
ABC transporter substrate binding lipoprotein [Streptococcus pneumoniae]	CJG46427.1
ABC-2 type transporter family protein [Streptococcus pneumoniae GA52612]	EJG83970.1
acyltransferase family protein [Streptococcus pneumoniae]	SNL85654.1
alanine aminotransferase, partial [Streptococcus pneumoniae]	KXW50488.1
alanyl-tRNA synthetase [Streptococcus pneumoniae]	CKG69103.1
aldo/keto reductase [Streptococcus pneumoniae]	WP_001269452.1
α-acetolactate decarboxylase [Streptococcus pneumoniae]	CJV90221.1
α-amylase [Streptococcus pneumoniae GA60080]	EJH11203.1
aminotransferase [Streptococcus pneumoniae GA11184]	EHD26622.1
anaerobic ribonucleotide reductase [Streptococcus pneumoniae]	CJA41997.1
cell wall surface anchor family protein [Streptococcus pneumoniae]	COK42161.1
chlorohydrolase [Streptococcus pneumoniae]	CYH35105.1
ecoEI R domain protein [Streptococcus pneumoniae GA13723]	EHZ24329.1
endo-α-N-acetylgalactosaminidase [Streptococcus pneumoniae]	CKF12265.1
exopolyphosphatase [Streptococcus pneumoniae GA47562]	EJH24984.1
glycosyl hydrolase family 20 (GH20) protein [Streptococcus pneumoniae]	CIV37094.1
glycosyl transferase [Streptococcus pneumoniae]	CKG70649.1
hlyIII superfamily protein [Streptococcus pneumoniae]	SNI42750.1
Holliday junction-specific endonuclease [Streptococcus pneumoniae]	SNH04621.1
hypothetical protein [Streptococcus pneumoniae]	WP_000842498.1
hypothetical protein [Streptococcus pneumoniae]	WP_050239143.1
hypothetical protein [Streptococcus pneumoniae]	WP_000977365.1
hypothetical protein CGSSpBS455_01575 [Streptococcus pneumoniae BS455]	EFL65997.1
hypothetical protein D059_00935	EOB20305.1
hypothetical protein D061_04916 [Streptococcus pneumoniae 1488]	EOB23299.1
hypothetical protein PNI0008_00610, partial [Streptococcus pneumoniae PNI0008]	ELU73227.1
hypothetical protein SP_1041 [Streptococcus pneumoniae TIGR4]	AAK75156.1
hypothetical protein, partial [Streptococcus pneumoniae]	WP_079098811.1
isoleucyl-tRNA synthetase [Streptococcus pneumoniae]	COK04924.1
membrane protein [Streptococcus pneumoniae]	CRH99656.1
oxidoreductase%2C pyridine nucleotide-disulfide class I%2C Mercury (II) reductase [Streptococcus pneumoniae]	CIV90663.1
penicillin binding protein 1A [Streptococcus pneumoniae]	WP_001040024.1
penicillin binding protein 2B, partial [Streptococcus pneumoniae]	BAA11616.1
phosphorylcholine transferase LicD [Streptococcus pneumoniae]	WP_078161399.1
PLP-dependent aminotransferase family protein [Streptococcus pneumoniae]	WP_050199184.1
PTS system IIABC components [Streptococcus pneumoniae]	COO88789.1
P-type ATPase-metal cation transport [Streptococcus pneumoniae]	CMW01002.1
sensor histidine kinase [Streptococcus pneumoniae]	WP_061753039.1
sialidase A (neuraminidase A) [Streptococcus pneumoniae]	COT00654.1
sugar ABC transporter permease, partial [Streptococcus pneumoniae]	WP_050272294.1
transcriptional regulator, GntR family [Streptococcus pneumoniae G54]	ACF56815.1
tryptophan synthase subunit α, partial [Streptococcus pneumoniae]	WP_085820027.1

Gene name	Sequence ID
TVP38/TMEM64 family protein [Streptococcus pseudopneumoniae]	WP_049513140.1
type 4 prepilin peptidase [Streptococcus pneumoniae]	CJD57248.1
UDP-N-acetylmuramyl tripeptide synthetase%2C Mur ligase [Streptococcus pneumoniae]	COH16933.1
Uncharacterized protein [Streptococcus pneumoniae]	SNK82086.1
Uncharacterized protein [Streptococcus pneumoniae]	CIV83149.1
YwnB [Streptococcus pneumoniae]	COG36585.1

Technical Appendix Table 5. WGS-based antimicrobial resistance detection and pilus determinant detection platform

Query (No. of bp)	Accession, sequence coordinates	Supplementary information
pbp1a (2160)	AE007317, 332863–335022 (complement)	Transpeptidase domain, 333083–333913
pbp2b (2057)	AE007317, 1494216–1496273 (complement)	Transpeptidase domain, 1494292–1495124
pbp2x (2253)	AE007317, 302261–304513	Transpeptidase domain, 302945–304019
ermB (100)	HG799494, 44520–44619	≧95% sequence identity predicts presence of the resistance gene
ermTR (68)	CP002121,856516–856583	≧95% sequence identity predicts presence of the resistance gene
mefA (1218)	U70055, 314–1531	≧98% sequence identity predicts presence of the resistance gene
mefE (1218)	U83667, 1–1218	≧98% sequence identity predicts presence of the resistance gene
tetM (100)	HG799494, 42545–42644	≧95% sequence identity predicts presence of the resistance gene
tetM (1935)	HG799494, 41018–42952	a deletion of two nucleotides at codon 339, generating a premature stop codon
tetO (100)	FM178797, 1754–1853 (complement)	≧95% sequence identity predicts presence of the resistance gene
folA (507)	AE007317,1412861–1413367 (complement)	I100L (common) and D92R substitutions confer trimethoprim resistance
folP (945)	AE007317, 268022–268966	various insertions of 1–2 codons between bases 168 and 201 of *folP* confer sulfamethoxazole resistance and intermediate cotrimoxazole resistance
rrgA-1 (100)	CP000921, 463577–463676	≧95% sequence identity predicts presence of pili-1
pitB-1 (100)	CP000921, 1003530–1003629	≧95% sequence identity predicts presence of pili-2

Technical Appendix Table 6. PBP1a transpeptidase domain sequences that were newly identified in this study

Sequences
>new1
SMKPITDYAPALEYGVYDSTASIVHDVPYNYPGTDTPLYNWDHVYFGNITIQYALQQSRNVTAVETLNKVGLDRAKTFLNGLGIDY<br>PSMHYANAISSNTTESNKKYGASSEKMAAAYAAFANGGIYHKPMYINKIVFSDGSEKEFSDAGTRAMKETTAYMMTEMMKTVLTY<br>GTGRGAYLPWLPQAGKTGTSNYTDEEIEKYIKNTGYVAPDEMFVGYTRKYSMAVWTGYSNRLTPIVGDGFLVAAKVYRSMITYLS<br>EDTHPEDWTMPDGLFRNGEFV
>new2
SMKPITDYAPALEYGVYDSTATIVHDEPYNYPGTDIPVYNWDRGYFGNITLQYALQQSRNVPAVETLNKVGLNRAKTFLNGLGIDY<br>PSLHYSNAISSNTTESDQKYGASSEKMAAAYAAFANGGTYYKPMYIHKVVFSDGSEKEFSNVGTRAMKETTAYMMTDMMKTVLT<br>YGTGRGAYLPWLPQAGKTGTSNYTDEEIEKYIKNTGYVAPDEMFVGYTRKYSMAVWTGYSNRLTPLVGNGLTVAAKVYRSMMTY<br>LSEGSNPEDWNIPEGLYRNGEFV
>new3
SMKPITDYAPALEYGVYDSTASIVHDVPYNYPGTDTPLYNWDHVYFGNITIQYALQQSRNVTAVETLNKVGLDRAKTFLNGLGIDY<br>PSMHYANAISSNTTESNKKYGASSEKMAAAYAAFANGGIYHKPMYINKIVFSDGSEKEFSDAGTRAMKETTAYMMTDMMKTVLSY<br>GTGRNAYLAWLPQAGKTGTSNYTDEEIEKYIKNTGYVAPDEMFVGYTRKYSMAVWTGYSNRLTPIVGDGFLVAAKVYRSMMTYL<br>SEGSNPEDWNIPEGLYRNGEFV
>new4
TMKPITDYAPAIEYGIYDSTATMVNDIPYNYPGTSTPVYNWDRAYFGNITLQYALQQSRNVPAVETLNKVGLDRAKNFLNGLGIDYP<br>DMHYSNAISSNTTESNKQYGASSEKMAAAFAAFANGGIYHKPMYINKIVFSDGSEKEFSDAGTRAMKETTAYMMTEMMKTVLSY<br>GTGRNAYLAWLPQAGKTGTSNYTDEEIENHIKTSQFVAPDELFAGYTRKYSMAVWTGYSNRLTPLVGNGLTVAAKVYRSMMTYL<br>SEGSNPEDWNIPEGLYRNGEFV
>new5
TMKPITDYAPAIEYGVYDSTATMVNDIPYNYPGTSTPVYNWDRAYFGNITLQYALQQSRNVPAVETLNKVGLDRAKNFLNGLGIDY<br>PDMHYSNAISSNTTESNKQYGASSEKMAAAFAAFANGGIYHKPMYINKIVFSDGSEKEFSDAGTRAMKETTAYMMTEMMKTVLSY<br>GTGRNAYLAWLPQAGKTGTSNYTDEEIENHIKTSQFVAPDELFVGYTRKYSMAVWTGYSNRLTPLVGNGLTVAAKVYRSMMTYL<br>SEGSNPEDWNIPEGLYRNGEFV

Technical Appendix Table 7. PBP2b transpeptidase domain sequences that were newly identified in this study

Sequences
>new1
TNVFVPGSVVKAATISSGWENGVLSGNQTLTDQPIVFQGSAPIYSWYKLAYGSFPITAVEALEYSSNAYMVQTALGIMGQTYQPN<br>MFVGTSNLESAMEKLRSTFGEYGLGTATGIDLPDESTGFVPKEYSFANYITNAFGQFDNYTPMQLAQYVATIANNGVRVAPRIVEG<br>IYGNNDKGGLGDLIQQLQPTEMNKVNISDSDMSILHQGFYQVAHGTSGLTTGRAFSNGAAVSISGKTGTAESYVEGGQEANNTNA<br>VAYAPSDNPQIAVAVVFPHNTN
>new2
TNVFVPGSVVKAATISSGWENGVLSGNQTLTDQPIVFQGSAPIYSWYKLAYGSFPITAVEALEYSSNAYMVQTALGIMGQTYQPN<br>MFVGTSNLETAMGKLRATFGEYGLGAATGIDLPDESTGFVPKEYSFANFITNAFGQFDNYTPMQLAQYVATIANNGVRLAPHIVEG<br>IYDNNDKGGLGELIQAIDTKEINKVNISESDMAILHQGFYQVSHGTSPLTTGRAFSDGATVSISGKTGTGESYVAGGQEANNTNAVA<br>YAPTENPQIAVAVVFPHNTN
>new3
TNVFAPGSVVKAATISSGWENGVLSGNQTLTDQSIVFQGSAPINSWYTQAYGSFPITAVQALEYSSNAYMVQTALGLMGQTYQPN<br>MFVGTSNLESAMGKLRSTFGEYGLGSATGIDLPDESTGFVPKDYSFANYITNAFGQFDNYTPMQLAQYVATIANDGVRVAPRIVE<br>GIYGNNDKGGLGDLIQQLQPTEMNKVNISDSDMSILHQGFYQVAHGTSGLTTGRAFSNGAAVSISGKTGTAESYVEGGQEANNTN<br>AVAYAPSDNPQIAVAVVFPHNTN
>new4
TNVFVPGSVVKAATISSGWENGVLSGNQTLTDQSIVFQGSAPINSWYTQAYGSFPITAVQALEYSSNAYMVQTALGLMGQTYQPN<br>MFVGTSNLESAMGKLRSTFGEYGLGSATGIDLPDESTGFVPKDYSFANYITNAFGQFDNYTPMQLAQYVATIANDGVRVAPRIVE<br>GIYGNNDKGGLGDLIQQLQPTEMNKVNVSDSDMSILHQGFYQVAHGTSGLTTGRAFSNGAAVSISGKTGTAESYVEGGQEANNT<br>NAVAYAPSDNPQIAVAVVFPHNTN

Technical Appendix Table 8. PBP2x transpeptidase domain sequences that were newly identified in this study

Sequences
>new1
GTDGIITYEKDRLGNIVPGTEQVSQQTVDGKDVYTTISSPLQSFMETQMDAFQEKVKGKYMTATLVSAKTGEILATTQRPTFDADT<br>KEGITEDFVWRDILYQSNYEPGSPMKVMMLAAAIDNNTFPGGEVFNSSELKIADATIRDWDVNEGLTGGRMMTFSQGFAHSSNV<br>GMTLLEQKMGDATWLDYLNRFKFGVPTRFGLTDEYAGQLPADNIVNIAMSAFGQGISVTQTQMLRAFTAIANDGVMLEPKFISALY<br>DPNDQSVRKSQKEIVGNPVSKEAASVTRDHMVMVGTDPTYGTMYNHSTGKATVNVPGQNVALKSGTAEIADEKNGGYLTGSTN<br>NIFSVVSMHPAENPDFILYV
>new2
GKDGIITYEKDRLGNIVPGTEQVSQQTVDGKDVYTTISSTLQSFMETQMDAFLEKVKGKYMTATLVSAKTGEILATTQRPTFNADTK<br>EGITEDFVWRDILYQSNYEPGSAMKVMTLASSIDNNTFPSGEYFNSSEFKIADATTRDWDVNDGLTTGGMMTFLQGFAHSSNVG<br>MSLLEQKMGDATWLDYLSRFKFGVPTRFGLTDEYAGQLPADNIVSIAQSSFGQGISVTQTQMLRAFTAIANDGVMLEPKFISAIYDT<br>NNQSVRKSQKEIVGNPVSKEAASTTRNHMILVGTDPLYGTMYNHYTGKPIITVPGQNVAVKSGTAQIADEKNGGYLVGSTNYIFSV<br>VTMNPAENPDFILYV
>new3
GKDGIITYEKDRLGNIVPGTEQVSQQTVDGKDVYTTLSSPLQSFMETQMDAFLEKVKGKYMTATLVSAKTGEILATTQRPTFNADT<br>KEGITEDFVWRDILYQSNYEPGSAFKVMMLASSIDNNTFPSGEYFNSSEFKIADATTRDWDVNEGLTTGGMMTFSQGFAHSSNV<br>GTSLLEQKMGDATWLDYLKRFKFGVPTRFGLTDEYAGQLPADNIVSIAQSSFGQGISVTQTQMLRAFTAIANDGVMLEPKFISAIYD<br>TNNQSVRKSQKEIVGNPVSKEAASTTRNHMILVGTDPLYGTMYNHYTGKPIITVPGQNVAVKSGTAQIADEKNGGYLVGSTNYIFS<br>VVTMNPAENPDFILYV
>new4
GTDGIITYEKDRVGNIVPGTELVSQQTVDGKDVYTTLSSPLQSFMETQMDAFLEKVKGKYMTATLVSAKTGEILATTQRPTFNADT<br>KEGITEDFVWRDILYQSNYEPGSAMKVMTLAASIDNNTFPSGEYFNSSEFKIADATTRDWDVNEGLTTGGMMTFLQGFAHSSNVG<br>MSLLEQKMGDATWLDYLKRFKFGVPTRFGLTDEYAGQLPADNIVSIAQSSFGQGISVTQTQMLRAFTAIANDGVMLEPKFISAIYDT<br>NNQSVRKSQKEIVGNPVSKEAASTTRNHMILVGTDPLYGTMYNHYTGKPIITVPGQNVAVKSGTAQIADEKNGGYLVGSTNYIFSA<br>VTMNPAENPDFILYV
>new5
GTDGIITYEKDRLGNIVPGTELVSQQTVDGKDVYTTLSSPLQSFMETQMDAFLEKVKGKYMTATLVSAKTGEILATTQRPTFNADT<br>KEGITEDFVWRDILYQSNYEPGSAMKVMTLASSIDNNTFPSGEYFNSSEFKIADATTRDWDVNEGLTTGGMMTFLQGFAHSSNVG<br>MSLLEQKMGDATWLDYLKRFKFGVPTRFGLTDEYAGQLPADNIVSIAQSSFGQGISVTQTQMLRAFTAIANDGVMLEPKFISAIYDT<br>NNQSVRKSQKEIVGNPVSKEAASTTRNHMILVGTDPLYGTMYNHYTGKPIITVPGQNVAVKSGTAQIADEKNGGYLVGSTNYIFSA<br>VTMNPAENPDFILYV
>new6
GKDGIITYEKDRLGNIVPGTEQVSQQTVDGKDVYTTLSSPLQSFMETQMDAFLEKVKGKYMTATLVSAKTGEILATTQRPTFNADT<br>KEGITEDFVWRDILYQSNYEPGSAFKVMMLASSIDNNTFPSGEYFNSSEFKIADATTRDWDVNAGLTTGGMMTFLQGFVHSSNVA<br>TSLLEQKMGDATWLDYLKRFKFGVPTRFGLTDEYAGQLPADNIVSIAQSSFGQGISVTQTQMLRAFTAIANDGVMLEPKFISAIYDT<br>NNQSVRKSQKEIVGNPVSKEAASTTRNHMILVGTDPLYGTMYNHYTGKPIITVPGQNVAVKSGTAQIADEKNGGYLVGSTNYIFSV<br>VTMNPAENPDFILYV
>new7
GTDGIITYEKDRLGNIVPGTEQVSQQTVDGKDVYTTLSSPLQSFMETQMDAFLEKVKGKYMTATLVSAKTGEILATTQRPTFNADT<br>KEGITEDFVWRDILYQSNYEPGSAMKVMTLASSIDNNTFPSGEYFNSSEFKIADATTRDWDVNAGLTTGGMMTFLQGFAHSSNVG<br>MSLLEQKMGDATWLDYLKRFKFGVPTRFGLTDEYAGQLPADNIVSIAQSSFGQGISVTQTQMLRAFTAIANDGVMLEPKFISAIYDT

Sequences
NNQSVRKSQKEIVGNPVSKEAASTTRNHMILVGTDPIYGTMYNHYTGKPIITVPGQNVAVKSGTAQIADEKNGGYLVGSTNYIFSV VTMNPAENPDFILYV

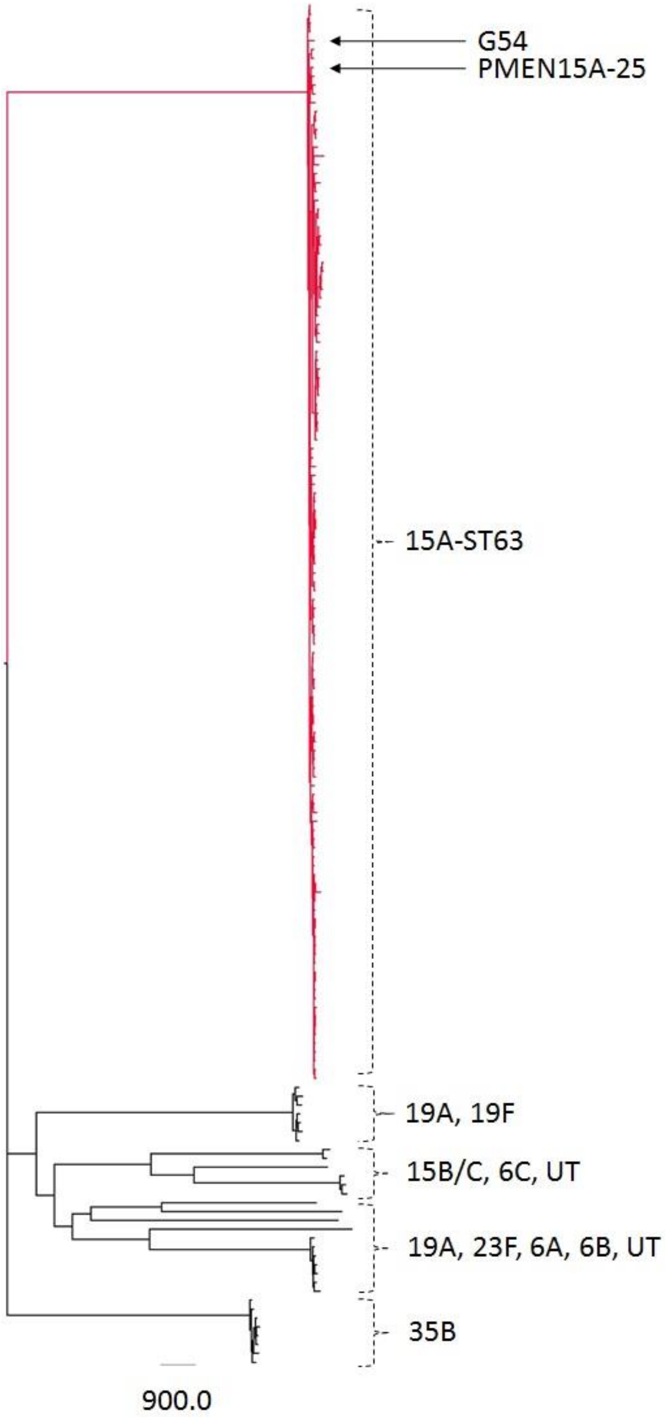

Technical Appendix Figure 1. The phylogenic tree was created using all Japanese and global isolates. All of the Japanese serotype 15A-sequence type (ST) 63 (meropenem-susceptible and meropenem-non-

susceptible) isolates were included in the same clade (red branch). None of the meropenem-non-susceptible serotype isolates except for 15A were included in the clade. This fact indicates that there was not an ancestral isolate of the meropenem-non-susceptible serotype 15A-ST63 isolate that had a serotype other than 15A within the tested isolates.

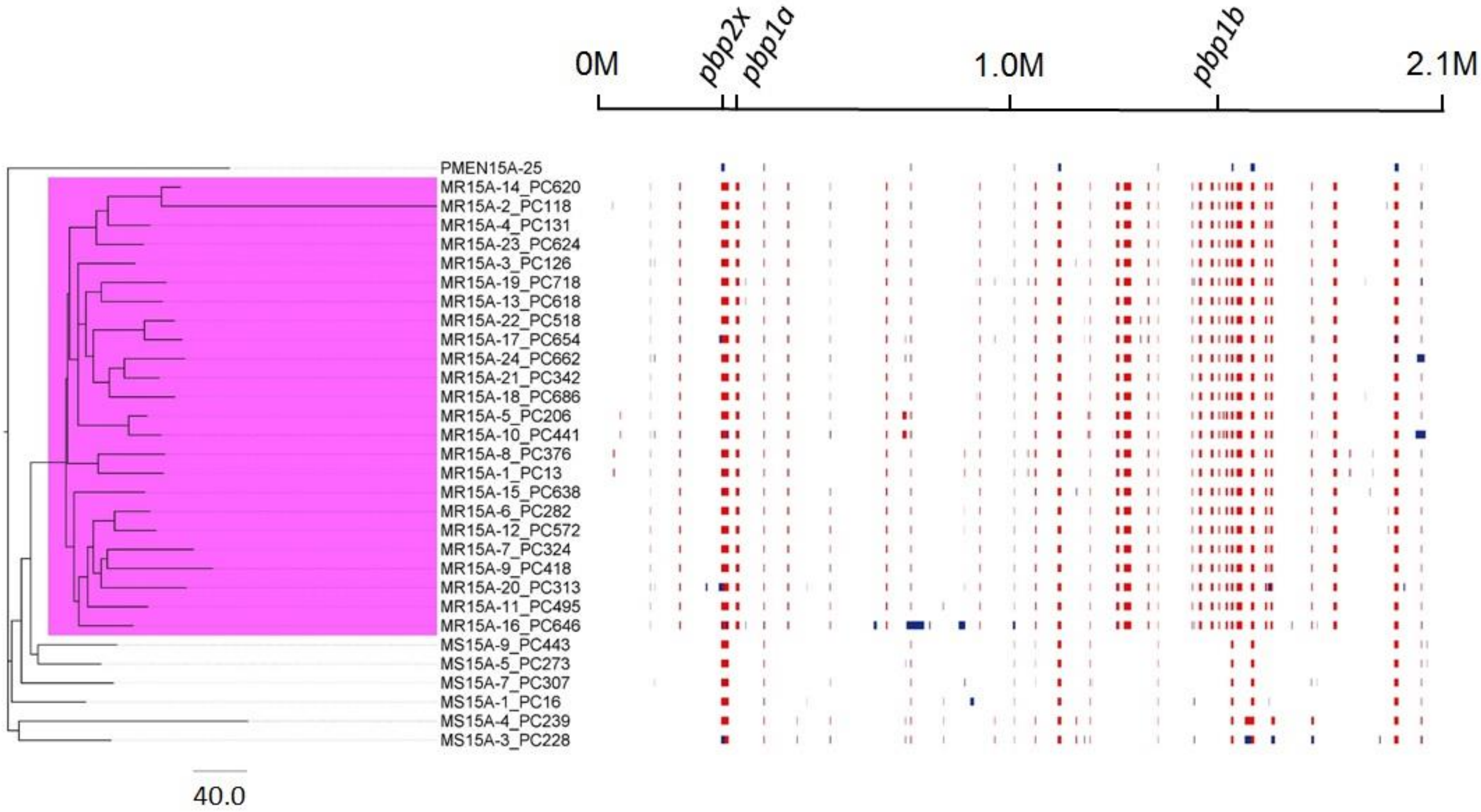

Technical Appendix Figure 2. The phylogenic tree created by Gubbins using all of the clade-I isolates (Figure 1) generated a Japanese meropenem-non-susceptible serotype 15A-sequence type (ST) 63-specific clade (highlighted in pink). All of the meropenem-non-susceptible serotype 15A-ST63 isolates were included in the clade, and none of the meropenem-susceptible serotype 15A-ST63 isolates were. In this analysis, PMEN15A-25 was used as an outgroup isolate. The block chart on the right shows the predicted recombination sites in each isolate. Blue blocks are unique to a single isolate, while red blocks are shared by multiple isolates.

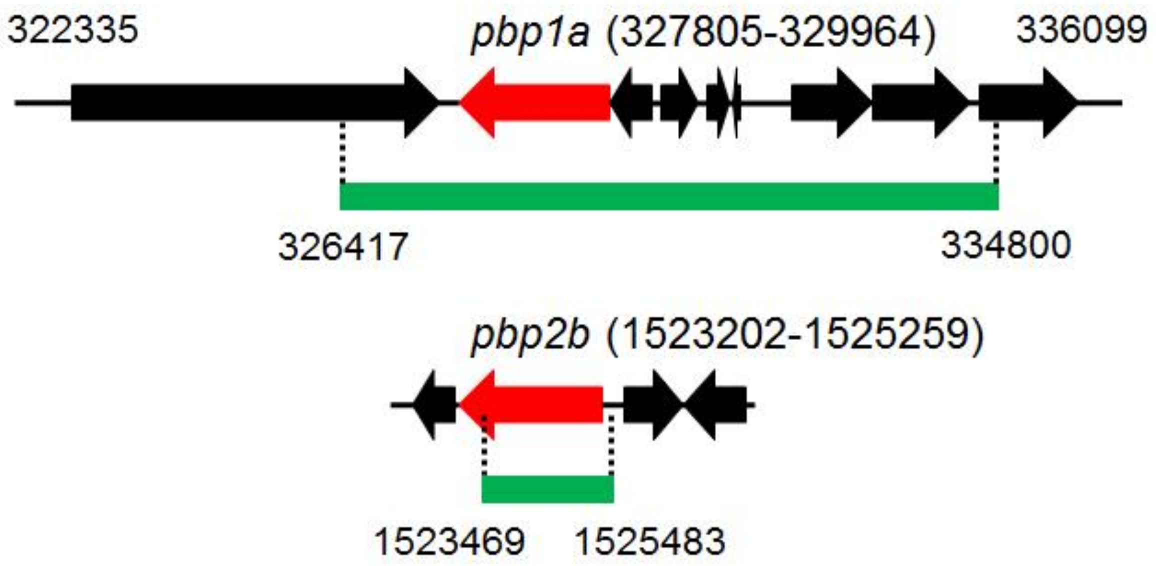

Technical Appendix Figure 3. Sketch of the predicted recombination sites including *pbp1a* and *pbp2b*, respectively. Green blocks show the recombination sites. Each number shows the sequence coordinates using *Streptococcus pneumoniae* G54 (NCBI Reference Sequence: NC_011072.11). These two recombination sites were shared by all of the meropenem-non-susceptible serotype 15A-ST63 isolates and were not found in any meropenem-susceptible serotype 15A-ST63 isolates.

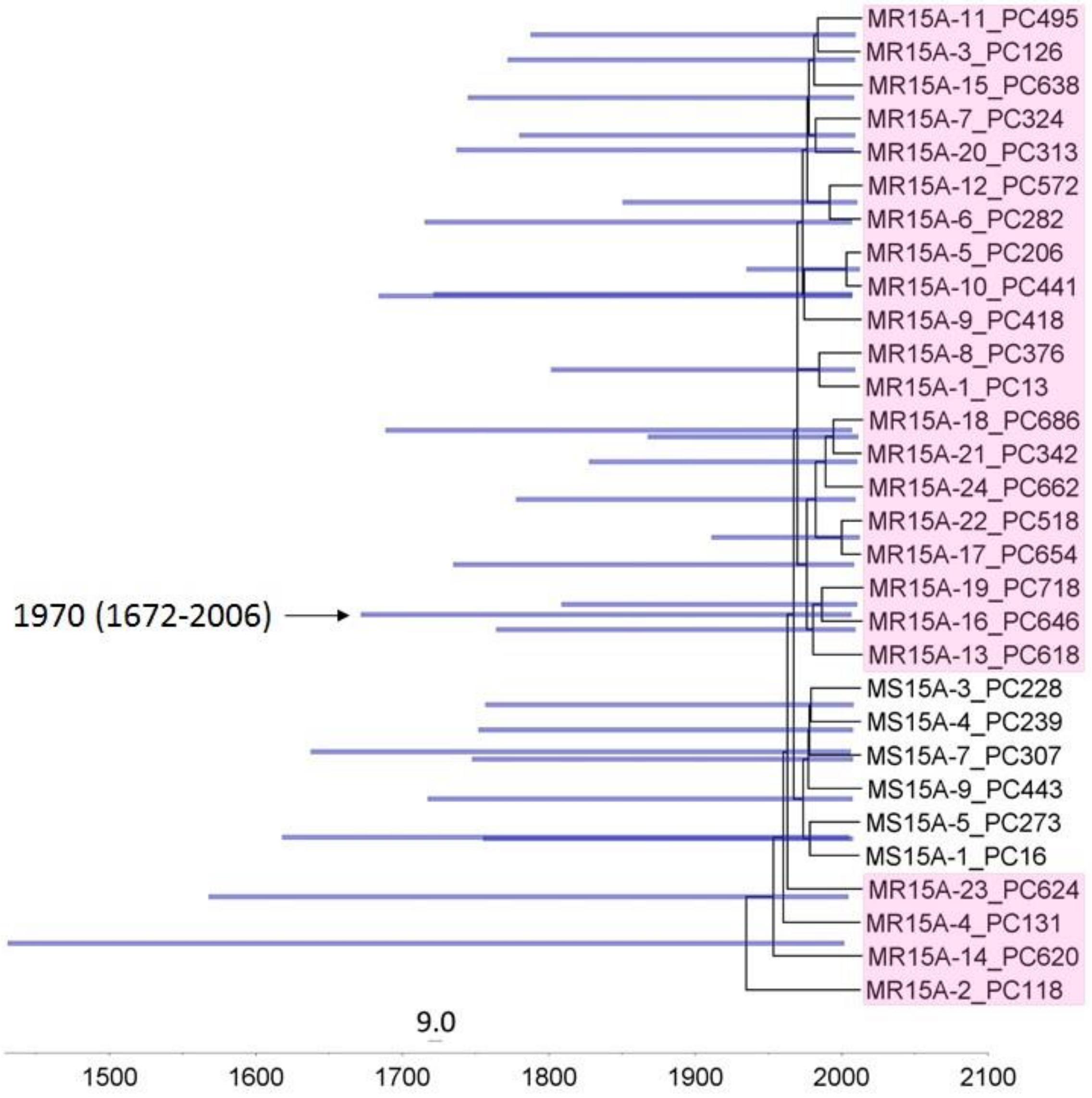

Technical Appendix Figure 4. The result of an estimation of the date at which meropenem-non-susceptible serotype 15A-ST63 originated. The “MR” (all of which are colored in pink) and “MS” prefixes in front of the isolate names indicate “Japanese meropenem-non-susceptible” and “Japanese meropenem-susceptible,” respectively. The blue bars showed the 95% credibility intervals. The blue bar indicated with an arrow is the target credibility interval at the node the MR- and MS-serotype 15A-ST63 strains were separated. The years to the left of the arrow show the node age and its 95% credibility interval.
